# A phylogenetic profiling approach identifies novel ciliogenesis genes in *Drosophila* and *C. elegans*

**DOI:** 10.15252/embj.2023113616

**Published:** 2023-06-15

**Authors:** Jeroen Dobbelaere, Tiffany Y Su, Balazs Erdi, Alexander Schleiffer, Alexander Dammermann

**Affiliations:** ^1^ Max Perutz Labs University of Vienna, Vienna Biocenter (VBC) Vienna Austria; ^2^ Vienna BioCenter PhD Program Doctoral School of the University of Vienna and Medical University of Vienna Vienna Austria; ^3^ Research Institute of Molecular Pathology, Vienna Biocenter (VBC) Vienna Austria; ^4^ Institute of Molecular Biotechnology of the Austrian Academy of Sciences, Vienna Biocenter (VBC) Vienna Austria

**Keywords:** *Caenorhabditis elegans*, centrioles, cilia, *Drosophila melanogaster*, evolution, Cell Adhesion, Polarity & Cytoskeleton, Development, Development, Methods & Resources

## Abstract

Cilia are cellular projections that perform sensory and motile functions in eukaryotic cells. A defining feature of cilia is that they are evolutionarily ancient, yet not universally conserved. In this study, we have used the resulting presence and absence pattern in the genomes of diverse eukaryotes to identify a set of 386 human genes associated with cilium assembly or motility. Comprehensive tissue‐specific RNAi in *Drosophila* and mutant analysis in *C. elegans* revealed signature ciliary defects for 70–80% of novel genes, a percentage similar to that for known genes within the cluster. Further characterization identified different phenotypic classes, including a set of genes related to the cartwheel component Bld10/CEP135 and two highly conserved regulators of cilium biogenesis. We propose this dataset defines the core set of genes required for cilium assembly and motility across eukaryotes and presents a valuable resource for future studies of cilium biology and associated disorders.

## Graphical abstract

Identification of a core set of genes associated with cilium assembly and motility across eukaryotes uncovers novel components of the basal body and regulators of ciliogenesis initiation.

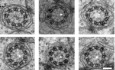

## Introduction

**Figure 1:**
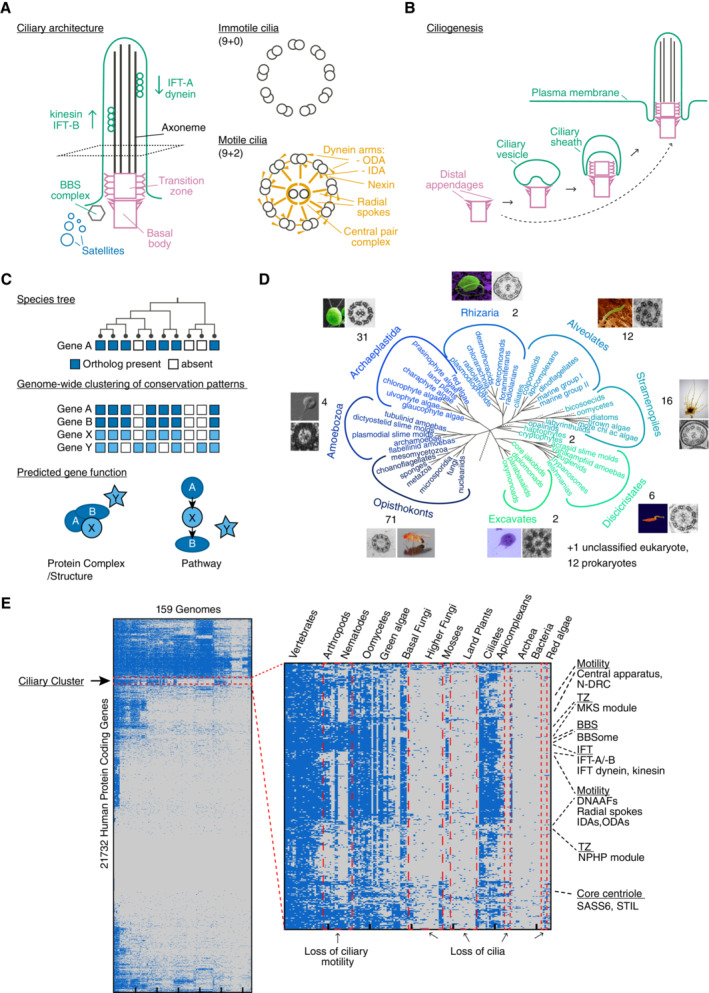
Identification of a ciliary cluster by phylogenetic profiling Schematic of ciliary architecture, depicting centriole‐derived basal body and transition zone (pink), BBSome and intraflagellar transport (IFT; green), axoneme (black) and centriolar satellites (blue). Cross sections illustrate axonemes of motile and immotile cilia. The latter display multiple additional features, including inner and outer dynein arms, nexins, radial spokes, and the central pair complex (yellow).Schematic of ciliogenesis process (based on Reiter *et al*, 2012). In the intracellular pathway, a vesicle forms in association with the basal body. The ciliary transition zone forms immediately distal to the basal body before the vesicle fuses with the plasma membrane, creating a ciliary pocket. IFT subsequently extends the ciliary axoneme beyond the transition zone. Alternatively, basal bodies dock directly to the plasma membrane prior to transition zone formation and axoneme elongation (dashed arrow). In *Plasmodium* and *Drosophila* sperm, axoneme elongation occurs in an IFT‐independent manner within the cytoplasm, whereupon the cilium is extruded from the cell (not depicted).Schematic of phylogenetic profiling approach (adapted from Dey *et al*, 2015). Reciprocal BLAST analysis using the human proteome as the starting point creates a presence and absence matrix for each gene across species. Genes functioning together in the same complex/structure or pathway share a similar inheritance pattern across evolution that is distinct from that of unrelated proteins, aiding in the identification of novel components (here: X).Eukaryotic tree (Baldauf, 2008), illustrating the presence of cilia in all major groups. One hundred and forty‐seven genomes from across eukaryotes as well as 12 prokaryotic species as outgroups were chosen for our phylogenetic profiling analysis. See Dataset EV1A. Image sources: Amoebozoa (*Phalansterium arcticum*, Shmakova *et al*, 2018), Archaeplastida (*Chlamydomonas reinhardtii*, Wolfram Weckwerth, Huang *et al*, 1979), Rhizaria (*Bigelowiella natans*, Paul Gilson and Geoff McFadden, Moestrup & Sengco, 2001), Alveolates (*Plasmodium falciparum*, Volker Brinkmann, Francia *et al*, 2015), Stramenopiles (*Ectocarpus siliculosus*, Delphine Scornet, Fu *et al*, 2013), Discicristates (*Trypanosoma brucei*, Thierry Blisnick and Philippe Bastin, Gluenz *et al*, 2015), Excavates (*Giardia lamblia*, National Institute of Infectious Diseases, Japan, Tůmová *et al*, 2021), and Opisthokonts (*Drosophila melanogaster*, André Karwath, this study).Phylogenetic profiling output, highlighting the ciliary cluster. Species nodes have been manually sorted to shift ciliated species as far to the left as possible while respecting the hierarchical clustering output. Gene order is unchanged and highlights the grouping of functional subcomplexes within the cluster. See also Dataset EV1B.

The highly stereotypical architecture of cilia and flagella (Fig 1A), terms often used interchangeably, can be found in all branches of the eukaryotic tree of life, albeit not in every species. Indeed, the number of cilia per cell was the defining characteristic to divide eukaryotes into two superclades, unikonts (literally: single flagellum) and bikonts (two; Cavalier‐Smith, 2002). Unikonts comprise the amoebozoa and opisthokonta (posterior flagellum), a clade that contains metazoa, fungi, and choanoflagellates, while bikonts comprise the remaining clades. Unikonts typically have only one cilium and associated basal body positioned at the posterior end of the cell, whereas bikonts have two, a younger, anterior, cilium and an older, posterior, cilium. Which of the two configurations represents the ancestral state is still unclear (Roger & Simpson, 2009), but what is clear is that the last common ancestor of all eukaryotes harbored both centrioles and cilia, while centriole‐organized centrosomes appear to be a much later opisthokont or metazoan innovation (Azimzadeh, 2014).

Cilia perform both sensory and motile functions. Ciliary motility is used to propel cells through fluid, as in the case of the flagellum of *Chlamydomonas* or vertebrate sperm, or fluid over the surface of the cell, as in the case of multiciliated epithelia of the vertebrate lung or oviduct. Ciliary motility usually involves dynein motor‐mediated ciliary bending (Viswanadha *et al*, 2017), although gliding motility involving ciliary adhesion to the substrate and internal intraflagellar transport (IFT)‐mediated transport of attachment sites has also been observed (Shih *et al*, 2013). Projecting from the cell surface and with a greatly enhanced surface‐to‐volume ratio, cilia are well suited to receive and transduce signals from the extracellular environment, including light, low‐molecular‐weight chemicals, proteins, and mechanical stimuli (Nachury, 2014). In vertebrates, exclusively nonmotile primary cilia are found on the surface of most cell types and are involved not only in the classical senses of vision and olfaction but are also integral to many of the signaling pathways that underlie development and tissue homeostasis (Satir & Christensen, 2007). The manifold functions of cilia in vertebrates are reflected in the pleiotropic nature of human cilium‐related disorders or ciliopathies, with clinical manifestations including *situs inversus*, respiratory dysfunction, infertility, and hydrocephalus for disorders affecting motile cilia, while disorders affecting nonmotile cilia are characterized by defects in neural tube development and limb patterning, cystic kidney, liver and pancreatic diseases, retinal degeneration, anosmia, cognitive defects, and obesity (Mitchison & Valente, 2017). Yet, while the most severe ciliopathies are perinatal lethal, they nevertheless represent only the weak end of the phenotypic spectrum, with complete loss of cilia resulting in embryonic lethality in mice (Murcia *et al*, 2000).

During ciliogenesis, centrioles dock either to a vesicle in the cytoplasm that fuses with the plasma membrane creating a ciliary pocket (intracellular pathway) or to the plasma membrane itself (extracellular pathway; Sorokin, 1962; Fig 1B). Central to this process are the transition fibers (Schmidt *et al*, 2012), which in vertebrates are derived from appendages present at the distal end of mature centrioles. These recruit a kinase, TTBK2, which promotes the removal of inhibitory factors present at the distal end of centrioles and the assembly of ciliary structures (Goetz *et al*, 2012). Formation of the outer microtubule doublets of the axoneme occurs by direct extension of centriolar microtubules, while the central pair of microtubules present in motile cilia forms *de novo* distal to the basal body (O'Toole *et al*, 2003). Motile cilia furthermore have multiple accessory structures decorating the axoneme (nexins, outer and inner dynein arms), which mediate ciliary bending (Ishikawa, 2017). Separating the basal body from the cilium proper is the transition zone, an elaborate structure characterized by Y‐links connecting axonemal microtubules to the ciliary membrane. The transition zone serves to restrict protein access, establishing the cilium as a distinct cellular compartment (Reiter *et al*, 2012). Extension of the axoneme within this compartment requires microtubule motor‐driven intraflagellar transport (IFT) delivering material to the ciliary tip (Kozminski *et al*, 1993).

Not all aspects of ciliogenesis are presently equally well understood. This applies in particular to the early steps involving docking of centrioles to the plasma membrane and initiation of transition zone assembly/axoneme extension. We have an increasingly detailed understanding of the molecular mechanisms underlying the intracellular pathway (Shakya & Westlake, 2021) in vertebrates. Yet, the degree to which these are relevant to the less well‐studied extracellular pathway is unclear, with key players apparently dispensable in this context and not conserved beyond metazoans (Ganga *et al*, 2021; Stuck *et al*, 2021). The role of centriolar appendages is also far from universal, with appendages seemingly lacking in *Drosophila* and *C. elegans* (Gottardo *et al*, 2015), without impairing basal body docking and initiation of ciliogenesis in these species. It is possible that different species have evolved different mechanisms for the same biological challenge. Alternatively, we do not yet possess the full set of conserved genes involved in the early steps of ciliogenesis. Missing genes likely also account for some of the still significant number of ciliopathy cases for which the causative genes have yet to be identified (Mitchison & Valente, 2017).

Our present compendium of ciliogenesis components (Vasquez *et al*, 2021) derives from a variety of different sources, including proteomics of isolated cilia in *Tetrahymena* (Smith *et al*, 2005) and *Chlamydomonas* (Pazour *et al*, 2005; Zhao *et al*, 2019) and genetic screens in *C. elegans* (Perkins *et al*, 1986), which led to the identification of much of the machinery for ciliary motility and IFT. Basal body and transition zone components in part have also been identified by proteomics (Andersen *et al*, 2003), but more commonly as the causative genes in human ciliopathies (Reiter & Leroux, 2017). Comprehensive genome‐wide RNAi or mutant screens have as yet not been carried out except in cultured cells (Kim *et al*, 2010; Wheway *et al*, 2015). Instead, targeted approaches have been employed to enrich for ciliary genes. Comparative genomics in particular has proven very fruitful. Two landmark studies published in 2004 took advantage of the first fully sequenced eukaryotic genomes to define ciliary genes by simple subtractive analysis (i.e., ciliated vs. nonciliated species), identifying key components of the ciliary trafficking machinery (Avidor‐Reiss *et al*, 2004; Li *et al*, 2004). More recently, the availability of increasing numbers of sequenced genomes has enabled more sophisticated phylogenetic profiling analyses, using co‐occurrence patterns across evolution to predict genes with a common function (Fig 1C; Pellegrini *et al*, 1999; Dey & Meyer, 2015). Here, we conducted a wider phylogenetic profiling approach, utilizing 147 genomes representing all major phyla of the eukaryotic tree to define a cluster of 386 human genes associated with the presence of cilia, including most of the known players involved in key steps of centriole assembly, cilium biogenesis, and motility, as well as another 152 genes that have so far not been functionally characterized. Systematic RNAi‐based analysis of novel genes in *Drosophila* as well as more targeted mutant analysis in *C. elegans* suggests that most if not all of these genes are likely cilium‐related, with our downstream characterization focusing on a set of genes involved in early steps of the ciliogenesis pathway.

## Results

### Identification of a ciliary cluster using a refined phylogenetic profiling approach

The present study emerged from a long‐standing interest in the laboratory in the molecular mechanisms underlying centriole assembly and function in ciliogenesis, using the nematode *Caenorhabditis elegans* and the fruit fly *Drosophila melanogaster* as experimental models (Dammermann *et al*, 2004, 2009; Serwas *et al*, 2017; Dobbelaere *et al*, 2020). Given the highly conserved nature of centrioles and cilia, we hypothesized that the early stages of ciliogenesis, including centriole to basal body conversion and initiation of axoneme extension, must also be conserved across eukaryotes. To identify candidate genes potentially involved in these processes we decided to apply a refined phylogenetic profiling approach. The original landmark studies had demonstrated the utility of comparative genomics approaches to identify ciliary genes (Avidor‐Reiss *et al*, 2004; Li *et al*, 2004). However, we noted that certain proteins such as the core centriolar components CENPJ/SAS‐4, STIL/SAS‐5/Ana2, and SASS6 tend to be missed in orthology inferences due to their high degree of sequence divergence making them drop below significance thresholds in rapidly evolving species such as *C. elegans* (Carvalho‐Santos *et al*, 2010; Hodges *et al*, 2010). In setting out to generate our phylogenetic profiles, we therefore chose a deliberately low BLASTp sequence similarity cutoff of 0.1, with bidirectional best match as a simple but robust (Kristensen *et al*, 2011) method to infer orthology. We further decided to use the human proteome as the starting point for our analysis, not only because it is the best annotated but also because vertebrates display a lower rate of sequence divergence compared with other eukaryotes (Douzery *et al*, 2004; Peterson *et al*, 2004; Raible *et al*, 2005). Phylogenetic profiling approaches have grown increasingly complex, with recent studies aimed at identifying ciliary genes incorporating information on the ciliary status of species, their evolutionary relationship, and/or training sets of verified ciliary genes, as well as iterative agglomerative clustering to identify orthogroups (Li *et al*, 2014; Dey *et al*, 2015; Nevers *et al*, 2017). We here opted instead for a simple hierarchical clustering approach, incorporating no information beyond the presence/absence pattern computed for each of the 21.732 human protein‐coding genes across the genomes of 147 diverse eukaryotes representing the different branches of the eukaryotic tree, as well as 12 prokaryotic species (9 archaea, 3 bacteria) as outgroups (see Fig 1D and Dataset EV1A). In selecting genomes, care was taken to avoid overrepresenting certain branches of the tree and to exclude incompletely sequenced/annotated genomes that would create false negatives in the phylogenetic analysis. Visual inspection of the clustering output revealed a set of 386 genes whose inheritance pattern was clearly distinct from that of other highly conserved genes (see Fig 1E and Dataset EV1B). Further analysis showed this set of genes to comprise key centriolar and ciliary genes, and their presence and absence pattern across eukaryotes to reflect the presence and absence of cilia in those species (see below). Such a single ciliary cluster had not been observed in previous analyses (Li *et al*, 2014; Dey *et al*, 2015; Nevers *et al*, 2017). We therefore sought to confirm the robustness of our analysis by reducing the number of species representing each branch of the tree by 25, 50, and 75%. A clear ciliary cluster could still be identified in all cases, although the percentage of cluster genes remaining dropped below 80% with the most aggressive perturbation (277/386 genes when using 42 genomes, see Dataset EV1). This is in line with published reports of diminishing returns in phylogenetic profiling approaches > 100 genomes (Škunca & Dessimoz, 2015). We conclude that our phylogenetic profiling approach robustly identifies a set of genes putatively linked to cilia.

### *In silico* characterization of the ciliary phylogenetic cluster

**Figure 2:**
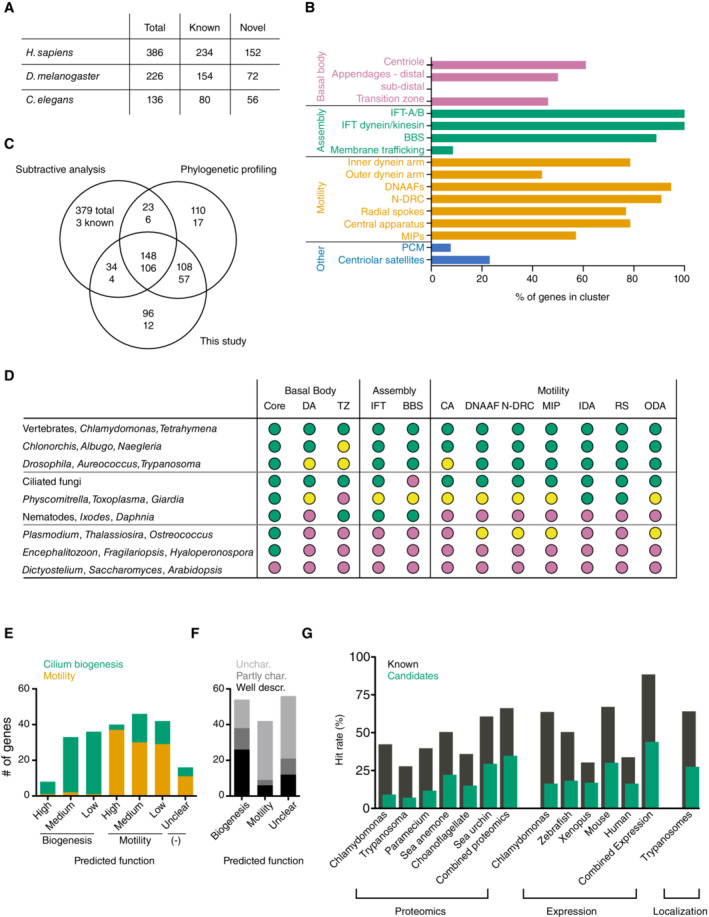
*In silico* analysis of the ciliary cluster Breakdown of ciliary cluster into known ciliary genes and those for which a ciliary function has yet to be established based on literature analysis. See also Dataset EV2A. Not all 386 genes are conserved in *Drosophila* and *C. elegans* given the extensive gene loss in ecdysozoa (Guijarro‐Clarke *et al*, 2020) and the additional loss of ciliary motility genes in nematodes.Percentage of known genes for different functional categories associated with centrosomes and cilia represented within the ciliary cluster. See Dataset EV2B for source data.Venn diagram comparing the present 386 gene ciliary cluster with the output of previous comparative genomics analyses. In each case, the first number represent the total number of genes in each category and the second any centrosomal/ciliary gene found in the combined lists of genes defined in (B). Subtractive analysis refers to the original comparative analyses performed in 2004 (Avidor‐Reiss *et al*, 2004; Li *et al*, 2004), combined into one dataset, phylogenetic profiling to the combined output of the more recent phylogenetic profiling studies (Li *et al*, 2014; Dey *et al*, 2015; Nevers *et al*, 2017).Hierarchical clustering of species based on known genes within the ciliary cluster from the indicated functional categories shown in (B) reveals nine distinct patterns of gene loss. Color code is green > 2/3 of genes in indicated category present, yellow > 1/3 of genes present, pink < 1/3 present. Representative species only shown in figure. See Dataset EV2C for full list and source data.Ciliary cluster genes can be sorted into predicted motility and biogenesis genes purely based on their pattern of conservation in species with motile and nonmotile cilia (see Methods). Graph compares predicted function (x‐axis) against actual, established function (color code on y‐axis) for all functionally characterized ciliary genes within the cluster (see annotation in Dataset EV2A), grouped by level of conservation of the gene in ciliated species. The relatively high frequency of incorrectly assigned biogenesis genes at lower levels of conservation reflects their loss in ecdysozoa unrelated to their specific ciliary function. No prediction could be made for genes present in <50% of ciliated species. See Dataset EV2D for full details.Predicted function of genes currently not linked to cilia based on phylogenetic footprint as in (E). Shading reflects overall level of functional characterization unrelated to any potential ciliary function.Graph showing percent of known and candidate genes within the cluster found in selected large‐scale proteomic, expression and localization studies. See Dataset EV2E for full details.

We next set out to examine the 386 genes in the cluster by performing a comprehensive literature and database analysis, including any information on their putative orthologs in nonvertebrate experimental models (Dataset EV2A). We classified each gene based on its level of functional characterization overall and any previously reported link to cilia on a scale of 0–3 (0—no information, 1—some indication of centriole/cilium‐related function, 2—confirmed function, and 3—clearly established molecular mechanism). We further set out to provide a short description of its proposed function, as well as any potential or confirmed ciliopathy association. At time of writing, 234 of the 386 genes in the cluster had a reported function in cilium assembly or motility (i.e., with a score of 2 or above), with the other 152 genes as yet functionally uncharacterized or not linked to cilia (Fig 2A). How comprehensive is this list for known ciliary complexes and structures? As shown in Fig 2B, more than 50% and in many cases nearer 100% of components reported to be associated with basal bodies (including centriolar and transition zone components), cilium assembly (IFT and BBS components), and motility (inner and outer dynein arm components and dynein assembly factors, nexins, N‐DRC, radial spoke and central apparatus components and the only recently identified microtubule inner proteins [MIPs]) can be found in the cluster, indicating essentially complete coverage of core components. Notably missing are components linked to centrosomes (PCM and centriolar satellites), subdistal appendages of centrioles and ciliary membrane trafficking, in line with the proposed recent evolutionary origin of centrosomes and the intracellular pathway of ciliogenesis discussed above (see also Dataset EV2B). Signaling genes (components of GPCR and Hh signaling pathways, TRP channel proteins) are likewise absent, reflecting the more recent association of those pathways with cilia in opisthokonts (Sigg *et al*, 2017). A comparison of the present cluster with the original comparative genomics studies (Avidor‐Reiss *et al*, 2004; Li *et al*, 2004) and more recent refined phylogenetic profiling approaches (Li *et al*, 2014; Dey *et al*, 2015; Nevers *et al*, 2017) revealed considerable overlap, but also components unique to one or other type of study (Fig 2C, Dataset EV2C). The enrichment of known ciliary components in the present study suggests our approach is similarly if not more effective at identifying *bona fide* components compared with other recent phylogenetic profiling approaches, although a direct comparison is complicated by the inclusion of known components as “seeds” in the latter analyses.

Of the 386 genes in the ciliary cluster, all but one can be found in two or more of the six major eukaryotic groups other than unikonts shown in Fig 1D (counting cryptophytes and haptophytes and disicristates and excavates each as one group). All genes therefore likely originate in the ancestral eukaryote and may have formed part of the original complement to assemble motile cilia. As discussed by David Mitchell in his 2016 review (Mitchell, 2016), diversification into modern organisms has involved not only acquisition of new features but also loss of existing ones, including part or all of the machinery to form cilia. Within the species represented in our analysis, cilia can be inferred to have been lost 13 times independently of each other over the course of evolution (4× in opisthokonts, 1× in amoebozoa, 3× in archaeplastidae, 2× in alveolates, 3× in stramenopiles, see Dataset EV1A), which helps explain the clear definition of the ciliary cluster in Fig 1E. Clustering species based on the major ciliary subcomplexes within the cluster reveals seven other patterns (Fig 2D and Dataset EV2C). The machinery for ciliary motility but not cilia themselves has been entirely lost twice (once in nematodes, once in the arthropod *Ixodes scapularis*) and once in part (in the crustacean *Daphnia pulex*). These and other losses (e.g., of BBSome components in ciliated fungi) give rise to the grouping of genes functioning together in ciliary subcomplexes seen in Fig 1E and can be used to predict the function of known genes based purely on their pattern of conservation with a high degree of accuracy (Fig 2E and Dataset EV2D). For the 152 novel candidate genes within the cluster, ~ 2/3 can be assigned as putative ciliary assembly or motility genes, while for the remaining 1/3 the phylogenetic footprint is insufficient to make a confident prediction (Fig 2F and Dataset EV2D). But is there any experimental evidence for a ciliary function for these genes? Indeed, there is. As summarized in Fig 2G, candidate genes have been identified in large‐scale proteomic, expression, and localization screens performed in different experimental models, albeit unsurprisingly (given that those selfsame screens were used to isolate and characterize ciliary genes) at a lower rate than known genes within the cluster (see also Dataset EV2E).

### Functional analysis of cluster genes reveals signature ciliary phenotypes in *Drosophila* and *C. elegans*

**Figure 3:**
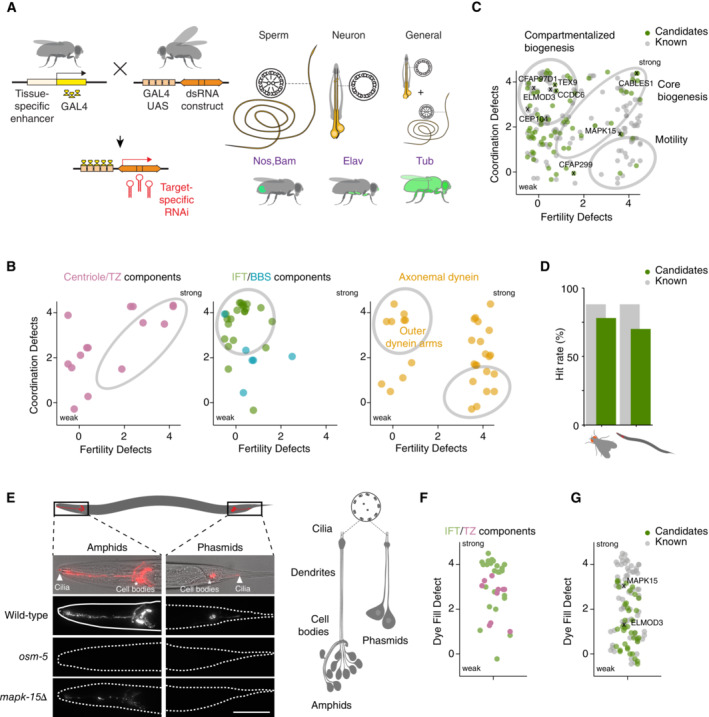
Results of primary screen in *C. elegans* and *Drosophila* In *Drosophila*, all conserved ciliary cluster genes were screened using the Gal4‐UAS system for tissue‐specific depletion by RNAi. Multiple RNAi lines per gene were crossed with drivers for neurons (Elav) and germline (Nanos and Bam combined), as well as a general driver (Tubulin). Flies were assayed for lethality, uncoordination and male fertility. Each phenotype was scored on a scale of 0 (no phenotype) to 4 (strong). See Dataset EV3A for definitions.Depletion of known centriolar and ciliary transition zone components results in defects in both coordination and male fertility, consistent with their core biogenesis function. In contrast, depletion of BBS and IFT components results in defects in neuronal function only, consistent with their role exclusively in compartmentalized biogenesis. Finally, depletion of axonemal dynein components primarily affects male fertility, although some outer arm dyneins also play significant roles in neuronal function. Each point on scatter plots represents one gene, with values shifted slightly (max ± 0.5) on both axes to avoid overlap of genes scoring identically.Scatter plot displaying the phenotypes of all known (gray) and previously uncharacterized genes (green) within the ciliary cluster, plotted as in (B). See Dataset EV3A for full details.Graphical summary of percentage of known (gray) and novel genes (green) within the ciliary cluster presenting significant (i.e. with a score > 1) ciliary phenotypes in *Drosophila* and *C. elegans*. Known and candidate genes scored similarly in both experimental models, suggesting a ciliary function for most if not all novel genes.In *C. elegans*, all conserved ciliary cluster genes with available mutants as well as genes of particular interest for which mutants were generated in the course of this study were screened using the dye‐fill assay to test for cilium structural integrity. Twelve amphid neurons in the head and four phasmid neurons in the tail, both featuring nonmotile sensory cilia, stain with the lipophilic dye DiI. Failure to take up dye is an indicator of ciliary structural defects. IFT mutants such as *osm‐5(p813)* tend to display dye‐fill null phenotypes, while other ciliary mutants present weaker phenotypes. Phenotypes were scored using a scale from 0 (no dye‐fill phenotype) to 4 (null). See Dataset EV3A for definitions.Scatter plot displaying the phenotypes of mutants in IFT and transition zone components. Each point on scatter plots represents one gene, with values shifted slightly to avoid overlap.Scatter plot displaying the phenotypes for all known (gray) and previously uncharacterized genes (green) within the ciliary cluster, plotted as in (F). See Dataset EV3A for full details. Data information: Scale bar is 50 μm (E). Source data are available online for this figure.

Based on our *in silico* analysis, we were confident that within this cluster we had a set of genes highly enriched in key players in cilium assembly and motility. To test whether candidate genes indeed have a cilium‐related function, we performed a comprehensive characterization by tissue‐specific RNAi in *Drosophila* and mutant analysis in *C. elegans*. *Drosophila* is an attractive experimental model for such questions in that it possesses multiple types of cilia, including motile sperm flagella that have been reported to form in an IFT‐independent manner in the cytoplasm, as well as sensory cilia that form in the canonical, IFT‐dependent manner in olfactory and mechanosensory neurons (Han *et al*, 2003; Jana *et al*, 2016). Candidate genes can therefore be easily classified into genes required for general and compartmentalized cilium biogenesis and cilium motility. Using the GAL4‐UAS system to deplete each candidate gene in the male germline (scoring male fertility), sensory neurons (assessing coordination in the negative geotaxis assay), and the whole animal (testing for nonspecific, pleiotropic phenotypes), we compared the resultant phenotypes to those for known ciliary genes within the cluster (Fig 3A–C). As can be seen in Fig 3B, centriolar and ciliary transition zone components presented moderately strong phenotypes in both tissues, consistent with their role in both canonical and compartmentalized cilium biogenesis (Basiri *et al*, 2014; Vieillard *et al*, 2016; Jana *et al*, 2018). In contrast, IFT components presented strong phenotypes almost exclusively in neurons, confirming and generalizing their reported role specifically in compartmentalized cilium biogenesis (Han *et al*, 2003). This was also true for the IFT‐associated BBSome, not previously functionally characterized in the fly, although phenotypes here were generally weak. In contrast, depletion of axonemal dyneins with the exception of certain outer arm dyneins strongly affected male fertility. Gravitaxis was also frequently perturbed, reflecting the role of dynein activity in mechanosensory neurons (Zur Lage *et al*, 2019). In total, 88% of known ciliary genes within the cluster presented significant phenotypes in neurons, sperm or both (Fig 3C and D, see also Dataset EV3A). Remarkably, this was true also for 54 of 69 novel genes (78%). Depleting candidate genes in the whole animal tended to reveal no additional phenotypes, ruling out broader cellular functions. Given the limitations of the assays employed in this primary screen, we conclude that most if not all novel candidate genes are indeed cilium‐linked.

To confirm and extend our findings, we turned to *C. elegans* as a complementary experimental model. Unlike *Drosophila*, *C. elegans* possesses exclusively nonmotile cilia, present in postmitotic sensory neurons, where they mediate the perception of chemosensory and mechanosensory stimuli. Compromised ciliary function leads to defects in complex behaviors such as chemotaxis, foraging, and male mating. However, critically, cilia are dispensable for viability and fertility, allowing mutants to be propagated in their homozygous state (Inglis *et al*, 2007). To investigate the potential role of candidate genes in cilium assembly, we took advantage of the dye‐fill assay as a simple and robust method to assess cilium structural integrity (Hedgecock *et al*, 1985). This assay is based on incubating worms with dilute solutions of the lipophilic dye DiI. In wild type, this dye is taken up by a subset of neurons (12 amphid neurons in the head, four phasmid neurons in the tail) through their exposed ciliary endings and accumulates in their cell bodies (Fig 3E). Defects in cilium assembly result in impaired dye‐filling, which can be easily scored under the fluorescence microscope. For known ciliary genes within the cluster, the dye‐fill assay revealed significant defects for 60 of 68 genes (88%), aided by the low animal‐to‐animal variability in *C. elegans*, with IFT mutants presenting stronger phenotypes than those affecting the transition zone (centriolar genes cannot be assayed given their essential role in embryonic development; Fig 3F and G, see also Dataset EV3A). Remarkably, 26 of 37 candidate genes (70%) likewise presented a dye‐fill phenotype. Phenotypes were generally weak, reflecting the fact that stronger, dye‐fill null mutants have been previously identified in exhaustive large‐scale screens using this assay (Perkins *et al*, 1986). With ecdysozoa and particularly nematodes having experienced widespread gene loss (Guijarro‐Clarke *et al*, 2020), including within the ciliary cluster, not all 386 genes could be assayed in *Drosophila* or *C. elegans*. Our results are, however, consistent with our hypothesis that essentially all cluster genes function in some aspect of cilium assembly or motility.

### Secondary analysis identifies candidate genes involved in the early stages of ciliogenesis

**Figure 4:**
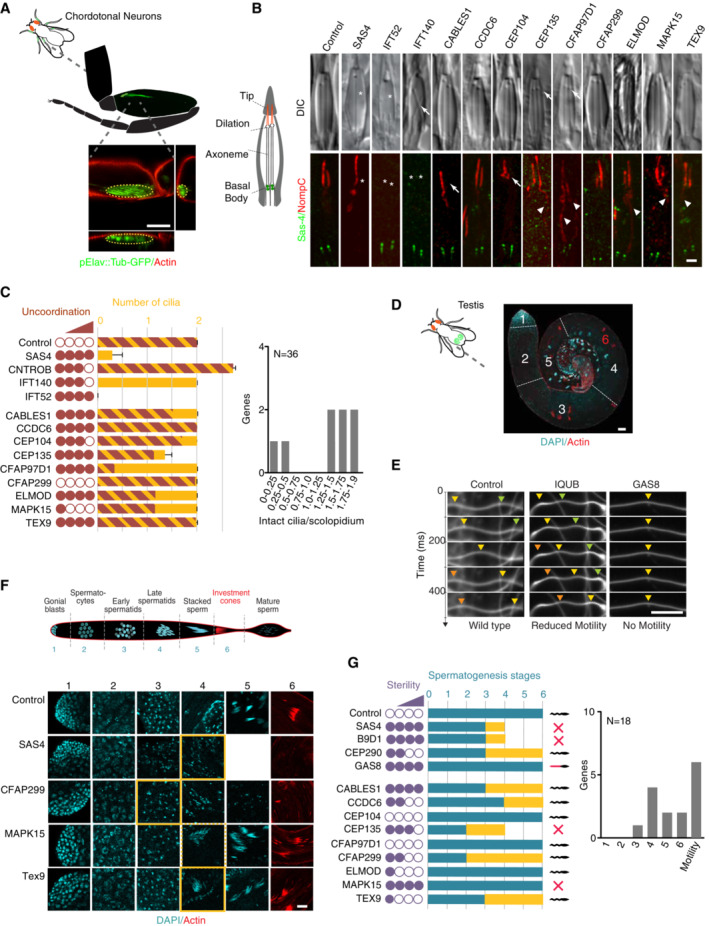
Results of secondary screen in *Drosophila* Schematic and confocal images of leg chordotonal neurons, visualized using pElav‐Gal4;UAS‐Tubulin‐GFP and actin staining, with xy, xz, and yz image projections to show position relative to the cuticle of the leg.Schematic and confocal microscopy images of scolopidia in chordotonal organ of the fly. Each scolopidium contains two cilia with their ends embedded in a cap cell. Scolopidium outline, cilia, and ciliary dilation can be visualized by DIC. Immunofluorescence micrographs with Sas‐4 and NompC antibodies used to mark basal bodies and ciliary tips, respectively. DIC images of flies depleted of candidate genes by RNAi reveal relatively few defects on par with IFT genes such as IFT140, with the exception of misplaced dilations for Cep135 and CFAP97D1 (arrows). Missing cilia (asterisks) as with SAS4 or IFT52 are not observed. NompC/Sas‐4 staining reveals more subtle phenotypes, including mislocalized NompC signal (with CFAP97D1, Cep135, Elmod, Mapk15, and Tex9, arrowheads) and apparent ciliary structural defects (Cep104 and Cables1, arrows). At least three animals examined per condition.Graph summarizing neuronal defects observed for candidate genes chosen for further analysis, with selected known centriolar/ciliary genes shown for comparison. Yellow bar is based on DIC analysis, brown bar based on NompC/Sas‐4. Uncoordination score is from primary screen. The second graph plots the number and severity of neuronal ciliary defects (DIC and NompC/Sas‐4 combined) for all previously uncharacterized ciliary genes chosen for further analysis. At least 12 scolopidia (DIC)/20 cilia (Sas‐4/NompC staining) from each of three different animals examined per condition.Image of dissected control testis stained for DNA and actin, showing the spatial separation of the different stages of spermatogenesis from stem cells (1) to bundles of mature sperm (5), each with their own distinct nuclear morphology. Also visible are the actin cones that strip away extra cytoplasm during the later stages of spermatogenesis.Mature sperm can be dissected from the seminal vesicle and motility analyzed using high‐speed video capture in dark‐field microscopy. Sinusoidal motion can be seen in wild type. In RNAi depletions of certain known motility genes (here IQUB and GAS8), this motion is reduced or even absent. Arrowheads mark position of individual bends in sequential frames. At least three movies made from different animals per condition.Spermatogenesis in wild‐type and RNAi depletions of selected genes, highlighting the stage where morphogenesis first becomes noticeably abnormal. At least six testes from three different animals examined per condition.Graph summarizing sperm defects observed for candidate genes chosen for further analysis, with selected known centriolar/ciliary genes shown for comparison. Yellow bar indicates stage where phenotypes first become apparent, and blue bar indicates furthest progression observed. Any sperm accumulating in the seminal vesicle were scored for motility. The second graph plots the severity of sperm ciliary defects (stage at which defects were first observed) for all previously uncharacterized ciliary genes chosen for further analysis. Number of animals examined as in (E, F). Data information: Scale bars are 50 μm (A, D), 1 μm (B), and 20 μm (E, F). Error bars in (C) are standard deviation. Source data are available online for this figure.

Although our primary screen in *Drosophila* allowed the identification and general classification of candidate genes, the precise nature of the process disrupted in each case remained to be determined. Thus, defects in male fertility could arise from a lack of sperm flagella or lack of sperm motility. Similarly, neuronal defects could arise from a lack of cilia or lack of mechanosensory function. To classify novel ciliary genes according to their function and identify candidates for further study, we employed a set of secondary assays for both sperm and neuronal cilia, comparing their depletion phenotypes to those for representatives of the various functional classes. For any gene scoring significantly in the coordination assays as well as those chosen for further characterization based on their spermatogenesis phenotype, the chordotonal organs responsible for proprioception located in the animals' legs (Fig 4A and B) were dissected and examined by DIC microscopy, as well as by immunofluorescence microscopy using antibodies to markers for the apical ciliary membrane (NompC) and basal bodies (Sas‐4; Dobbelaere *et al*, 2020). Chordotonal organs are made up of multiple scolopidia, each of which contains a pair of ciliated nerve endings ensheathed by a glial cell and attached with their ciliary tips to the cuticle via a cap cell (Kernan, 2007). We found defects in cilium biogenesis to manifest themselves in different ways, with depletion of centriole biogenesis and IFT components resulting in the total absence or reduced numbers of cilia, while depletion of other components yielded more subtle defects including shorter (as assessed by the position of the ciliary dilation) or morphologically abnormal cilia (Fig 4B). Depletion of candidate genes primarily resulted in the latter type of defect, with eight of 36 genes examined displaying shortened or abnormal cilia (selected examples shown in Fig 4B and C, quantitation in Dataset EV3B). Supernumerary cilia, formed by conversion of daughter centrioles into mothers, a phenotype associated with depletion of Centrobin (Gottardo *et al*, 2015), were not observed for any candidate gene.

For any gene scoring significantly in the male fertility assay as well as those chosen for further characterization based on their neuronal phenotype, the process of spermatogenesis was examined in two ways. First, testes were dissected, fixed, and stained to visualize DNA and the actin cytoskeleton. This allowed us to monitor the different stages of sperm differentiation using nuclear morphology, as well as the formation of actin cones during individualization (Fig 4D). Second, we dissected the seminal vesicle where mature sperm are stored and filmed the movement of sperm tails using dark field microscopy (Fig 4E). Examining known ciliary genes, we found that centriolar and transition zone components gave the strongest phenotypes, arresting sperm morphogenesis at early stages of differentiation, whereas motility genes only affected later stages of differentiation (Fig 4F and G). Interestingly, sperm found in the seminal vesicle were generally found to be at least partly motile, with defects in ciliary motility primarily manifesting themselves in an empty seminal vesicle as sperm fail to move from the testes to this storage organ. Depletion of candidate genes resulted in a wide spectrum of phenotypes, with some genes resulting in defects as early as the early spermatid stage, similar to Sas4 and CEP290, while others progressed as far as the individualization stage. Overall, 15 of 18 genes examined yielded significant phenotypes in this assay (Fig 4F and G, quantitation in Dataset EV3B).

In total, 19 of 37 genes (51%) examined based on their primary screen phenotype presented clear defects in either neurons or sperm. In our further characterization, we focused on a handful of those genes which presented particularly strong phenotypes consistent with a function in early stages of ciliogenesis and/or displayed an evolutionary conservation pattern consistent with a central role in cilium biogenesis.

### CABLES1, CCDC6, and TEX9 as novel basal body components related to Bld10/CEP135

**Figure 5:**
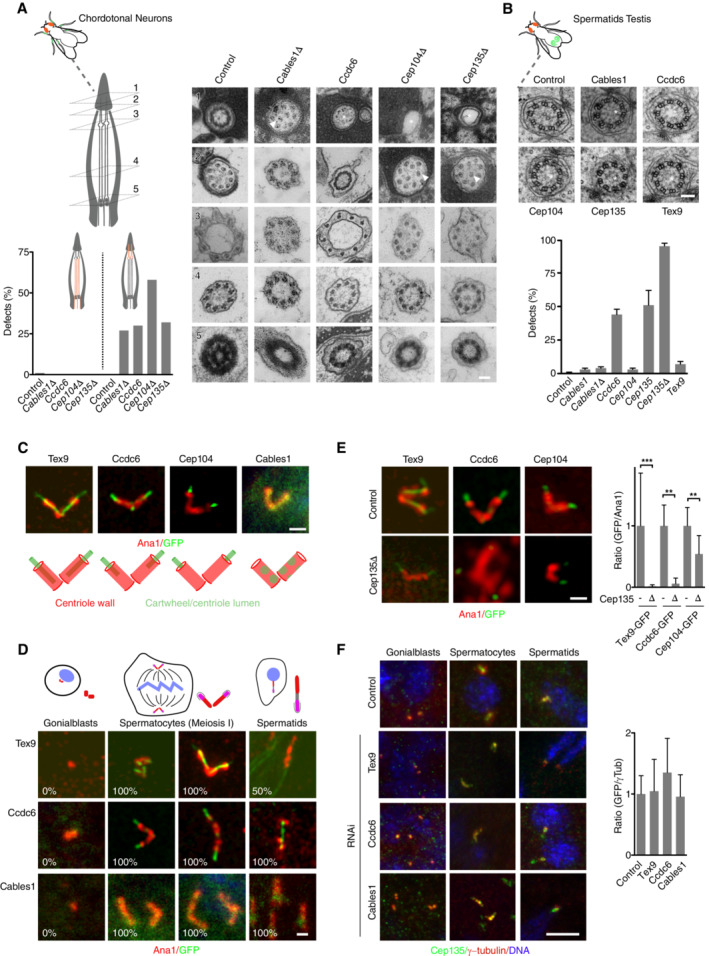
CABLES1, CCDC6, and TEX9 as novel basal body components related to Bld10/CEP135 A, B
RNAi‐mediated depletion/deletion of CABLES1, CCDC6, CEP104, and TEX9 in *Drosophila* chordotonal neurons (A) and sperm (B) results in defects in ciliary ultrastructure similar to CEP135. In neurons, there is a loss of ninefold symmetry toward the ciliary tips with displaced doublet microtubules (arrowheads) and axonemes frequently fail to reach the cap cell (asterisks). In sperm, there is a loss of the inner pair of microtubules (asterisks), a phenotype hitherto only associated with CEP135. At least three animals examined per condition and tissue.C
GFP fusions to TEX9, CCDC6, and CEP104 localize to the central lumen of sperm basal bodies marked by Ana1, while CABLES1 only faintly localizes to basal bodies.D
TEX9, CCDC6, and CABLES1 are recruited to maturing basal bodies during *Drosophila* spermatogenesis. *n* > 10 basal body pairs for each developmental stage.E, F
Localization interdependencies between CEP135, CEP104 and the novel components TEX9, CCDC6, and CABLES1 in spermatogenesis. CEP135 is required for the localization of TEX9, CCDC6, and CEP104 to mature basal bodies (E), while CEP135 localization is independent of TEX9, CCDC6, and CABLES1 at all stages of spermatogenesis (F). *n* > 6 basal body pairs per condition. Asterisks indicate statistically significant differences to the respective control (*t*‐test, **P < 0.01, ***P < 0.001). Data information: Scale bars are 100 nm (A, B), 1 μm (C–E), 5 μm (F). Error bars in (B, E, F) are standard deviation. Source data are available online for this figure.

Among the candidate genes in the screen that caught our attention were CABLES1, CCDC6 and TEX9, which presented significant phenotypes in neurons as well as sperm in our primary and secondary analyses (information on all genes further characterized in this study is collated in Dataset EV3C). TEX9 has previously been localized to centriolar satellites and the ciliary base in vertebrate cells (Gupta *et al*, 2015; Nevers *et al*, 2017), but remains functionally uncharacterized. CCDC6 has an extensive literature as a recurrent fusion partner for the oncogenic tyrosine kinases ROS1 and RET (Cerrato *et al*, 2018), yet itself remains entirely uncharacterized, although interestingly it was reported among the interactors in pulldowns of the IFT‐B component Cluap1/IFT38 (Beyer *et al*, 2018). Finally, CABLES1 has been reported to interact with CDK5 and c‐ABL and linked to control of cell proliferation and/or differentiation (Zukerberg *et al*, 2000; Wang *et al*, 2010), with no apparent connection to centrosomes or cilia. Given the severity of the neuronal and spermatogenesis phenotypes, ultrastructural analysis was performed on RNAi‐depleted animals (and in the case of CABLES1 also deletion mutants) for all three candidate genes. In the case of neuronal cilia, we observed a general disorganization and shortening of ciliary axonemes, with missing and displaced doublet microtubules toward the ciliary tip (Fig 5A). More striking was the phenotype in sperm, where the central pair of microtubules was frequently missing, while the outer microtubule doublets were unaffected (Fig 5B). Such a phenotype has hitherto been reported in *Drosophila* only for a single gene, the centriolar cartwheel component Bld10/CEP135 (Carvalho‐Santos *et al*, 2012). A re‐examination of CEP135 by RNAi and mutant analysis confirmed these earlier observations but also revealed defects in mechanosensation and ciliary ultrastructure in chordotonal neurons, similar to CABLES1 and CCDC6 (Fig 5A). We should note that the latter finding stands in contrast to initial reports on this gene in the fly which reported no apparent defects in coordination (Mottier‐Pavie & Megraw, 2009), which we attribute to the use of a stronger loss of function mutant than in the original work.

This phenotypic similarity to CEP135 prompted us to examine the localization of our novel components in the fly, with GFP transgenic fusions expressed under the general Ubq promoter. Remarkably, GFP fusions for both TEX9 and CCDC6, like CEP135 (Tian *et al*, 2021), localized to the center of the centriole barrel marked by Ana1, but extending further along the length of the extended spermatocyte basal body (Fig 5C). The third protein, CABLES1, also localized to spermatocyte basal bodies, albeit much more weakly, precluding detailed analysis. No localization was observed to centrosome‐organizing centrioles elsewhere in the fly. Instead, all three proteins were recruited as centrioles convert into basal bodies during spermatogenesis (Fig 5D). In the course of our studies, we came across one other known ciliary cluster gene, CEP104, which shared some of the phenotypic features of the above‐named components. Ultrastructural analysis of both RNAi‐depleted and mutant animals revealed the same signature ciliary defects in sperm and neurons (Fig 5A and B). CEP104 in vertebrates and *Chlamydomonas* has been shown to be a microtubule plus‐end tracking protein that translocates during ciliogenesis from the distal end of centrioles to the ciliary tip where it contributes to stability particularly of the central pair of microtubules (Jiang *et al*, 2012; Satish Tammana *et al*, 2013; Louka *et al*, 2018; preprint: Legal *et al*, 2023). Cep104 in *Drosophila* shares this localization, forming a central focus at the distal end of sperm basal bodies that co‐localizes with the most distal populations of CCDC6 and TEX9 (Fig 5C).

We then have four (with CABLES1 potentially five) components that share a localization to the core of basal bodies, with CEP135 at the proximal end and CEP104 at the distal end, and function in ciliogenesis, particularly in formation of the central pair and organization of ciliary tips. We therefore propose that these components form part of a common ciliogenesis pathway with CEP135 at its base. Consistent with such a pathway, loss of CEP135 is independent of CABLES1, CCDC6, and TEX9 (Fig 5F), but strongly affects the localization of all other components without affecting centriole assembly itself (Fig 5E).

### CFAP97D1 as part of a family of CFAP97 domain‐containing proteins required for axoneme assembly/stability

**Figure 6:**
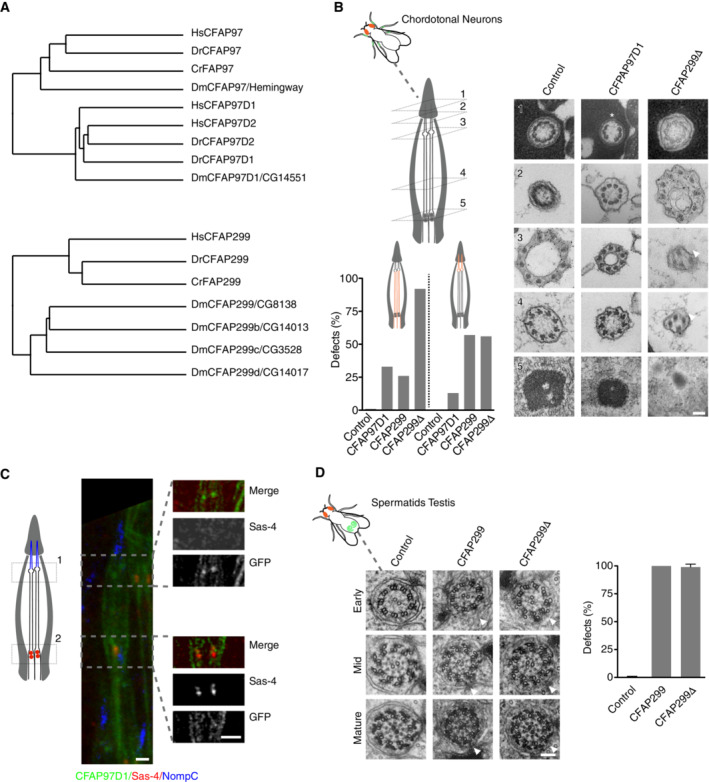
Conserved role for CFAP97 and CFAP299 family members in axoneme assembly and ciliogenesis Relationship between CFAP97 and CFAP299 family members in humans (Hs), zebrafish (Dr), *Chlamydomonas* (Cr), and *Drosophila* (Dm). Average distance trees calculated using the BLOSUM62 substitution matrix. *Drosophila* CFAP97D1 and its vertebrate paralogs CFAP97D1 and CFAP97D2 are distantly related to CFAP97/Hemingway, while CFAP299 has four paralogs in *Drosophila* with distinct tissue expression patterns. Characterized here is the most closely related ortholog, CG8138, hereafter CFAP299. Accession numbers: HsCFAP97 (NP_065878.1); DrCFAP97 (NP_997943.1); CrFAP97 (XP_042919651.1); DmCFAP97/Hemingway (NP_650714.1); HsCFAP97D1 (NP_001129955.1); HsCFAP97D2 (XP_016876399.1); DrCFAP97D1 (XP_697280.2); DrCFAP97D2 (XP_005167838.1); DmCFAP97D1/CG14551 (NP_651462.1); HsCFAP299 (XP_047305931.1); DrCFAP299 (NP_001108596.1); CrFAP299 (XP_001697404.2); DmCFAP299/CG8138 (NP_650260.1); DmCFAP299b/CG14013 (NP_608941.1); DmCFAP299c/DmCG3528 (NP_608686.1); DmCFAP299d/DmCG14017 (NP_608942.1).(B). RNAi‐mediated depletion/deletion of *Drosophila* CFAP97D1 and CFAP299 results in defects in ciliary ultrastructure in chordotonal neurons, including broken axonemes (asterisk) and perturbed ninefold symmetry (arrowheads). At least three animals examined per condition.A GFP fusion to CFAP97D1 localizes to the ciliary dilation but not basal body in chordotonal neurons. Insets acquired in Airyscan mode.RNAi‐mediated depletion/deletion of CFAP299 results in failure of membrane deposition around the developing sperm axoneme (arrowheads). At least three animals examined per condition. Data information: Scale bars are 100 nm (B, D), 1 μm (C). Error bars in (D) are standard deviation. Source data are available online for this figure.

CG14551/CFAP97D1 drew our attention due to its strong phenotype specifically in neurons. CG14551 is the only *Drosophila* ortholog of vertebrate CFAP97D1 and 2, part of a family of proteins that also includes CFAP97/KIAA1430 (Hemingway in *Drosophila*; Fig 6A). CFAP97D1 in mice has been found to be required for sperm flagellar axoneme integrity and male fertility (Oura *et al*, 2020), while its paralog CFAP97D2 remains uncharacterized. Similarly, Hemingway was found to be required for sperm flagellum assembly and ciliary motility in the fly, including in auditory sensory neurons, but with no apparent defects in coordination (Soulavie *et al*, 2014; vertebrate CFAP97 is presently uncharacterized). It was then surprising to see a phenotype for CFAP97D1 exclusively in neurons. Further characterization including by mutant analysis confirmed this specificity. Ultrastructural analysis of chordotonal cilia revealed broken axonemes and missing axonemal microtubule doublets, particularly toward the ciliary tip (Fig 6B), a phenotype reminiscent of what has been observed for sperm flagella with vertebrate CFAP97D1 and *Drosophila* Hemingway (Soulavie *et al*, 2014; Oura *et al*, 2020). CFAP97D1 was not localized in that study, while staining for Hemingway revealed no apparent localization to mature sperm. In contrast, *Drosophila* CFAP97D1 showed a clear ciliary localization, particularly in the area of the ciliary dilation toward the tip of chordotonal cilia (Fig 6C). Such a localization is consistent with the proposed function of CFAP97 domain‐containing proteins in cilium elongation/stability. Our data suggest that this function is not specific to motile cilia/flagella. Consistent with this, the Human Protein Atlas (proteinatlas.org, Uhlén *et al*, 2015) notes that while CFAP97D1 is testis specific, CFAP97D2 is more broadly expressed in ciliated tissues, including those bearing exclusively nonmotile cilia such as the pancreas, while CFAP97 is essentially ubiquitous.

### Unique role for CFAP299 in ciliary membrane deposition and ciliogenesis

CG8138/CFAP299 along with CG14013, CG14017, and CG3528 is one of four *Drosophila* orthologs of vertebrate C4orf22/CFAP299 (Fig 6A), a largely uncharacterized protein reported to be testis‐enriched and tentatively linked to spermatogenesis (Li *et al*, 2019a), but according to the Human Protein Atlas also expressed in other ciliated tissues including the lung and oviduct. CG8138 initially presented with a strong phenotype in male fertility in the primary and secondary screen. Ultrastructural analysis of both RNAi‐depleted and mutant animals revealed what to our knowledge is a unique and unprecedented phenotype: a seemingly intact flagellar axoneme but a total failure of membrane deposition to complete sperm individualization (Fig 6D). As described above, *Drosophila* spermatogenesis is highly unusual in that flagella form in an IFT‐independent manner in the cytoplasm and are then extruded in a process reminiscent of what is believed to occur in the malarial parasite *Plasmodium* (Sinden *et al*, 1976; Han *et al*, 2003). The proteins responsible for axoneme elongation in the cytoplasm, whether unique to that context or shared with canonical compartmentalized ciliogenesis, are not known. Tantalizingly, CFAP299 is also conserved in *Plasmodium*, yet its wider phylogenetic distribution, including in humans, suggests a function also in canonical ciliogenesis. Indeed, CG8138/CFAP299 mutant animals also presented severe defects in chordotonal neurons, with highly aberrant axonemes lacking ninefold symmetry (Fig 6B). CG8138 shares extensive homology with its paralogs CG14013, CG14017, and CG3528, also putatively expressed in neurons and sperm, raising the possibility of functional redundancy. Consistent with this, weaker phenotypes were also observed for CG14013 and CG3528 (see Dataset EV3A). How CFAP299 and its orthologs contribute to ciliogenesis is as yet unclear. No specific localization was observed for N‐ and C‐terminal GFP fusions in either sperm or neurons. However, its presence in the *Chlamydomonas* flagellar proteome (Pazour *et al*, 2005) suggests a role directly in cilium extension.

### MAPK15 and ELMOD as highly conserved regulators of ciliogenesis

The final two genes, MAPK15 and ELMOD, have to different degrees been previously characterized, but were nevertheless chosen for further analysis based on the type of protein they encode, their highly conserved nature and the severity of the phenotype associated with their depletion/mutation. MAPK15 (also known as ERK7 or 8) is an atypical mitogen‐activated protein kinase, atypical in the sense that it does not function as part of a classical three‐tiered MAPKKK–MAPKK–MAP kinase cascade (Coulombe & Meloche, 2007). Originally linked to cellular homeostasis and the maintenance of genomic integrity (Deniz *et al*, 2022), MAPK15 was more recently found to also regulate ciliogenesis by promoting the apical migration of basal bodies in *Xenopus*, reportedly by phosphorylating the actin regulator CapZIP (Miyatake *et al*, 2015). In *C. elegans*, putative *mapk‐15* loss of function mutations were found to perturb neuronal morphogenesis (McLachlan *et al*, 2018) but also ciliary architecture as well as the localization of many ciliary proteins (Kazatskaya *et al*, 2017; Piasecki *et al*, 2017). Ciliogenesis was also reported to be affected in human RPE1 cells (Kazatskaya *et al*, 2017). With the kinase localizing to the ciliary base, MAPK‐15 was proposed to act in a gating capacity to regulate ciliary trafficking and thereby ciliogenesis (Kazatskaya *et al*, 2017).

**Figure 7:**
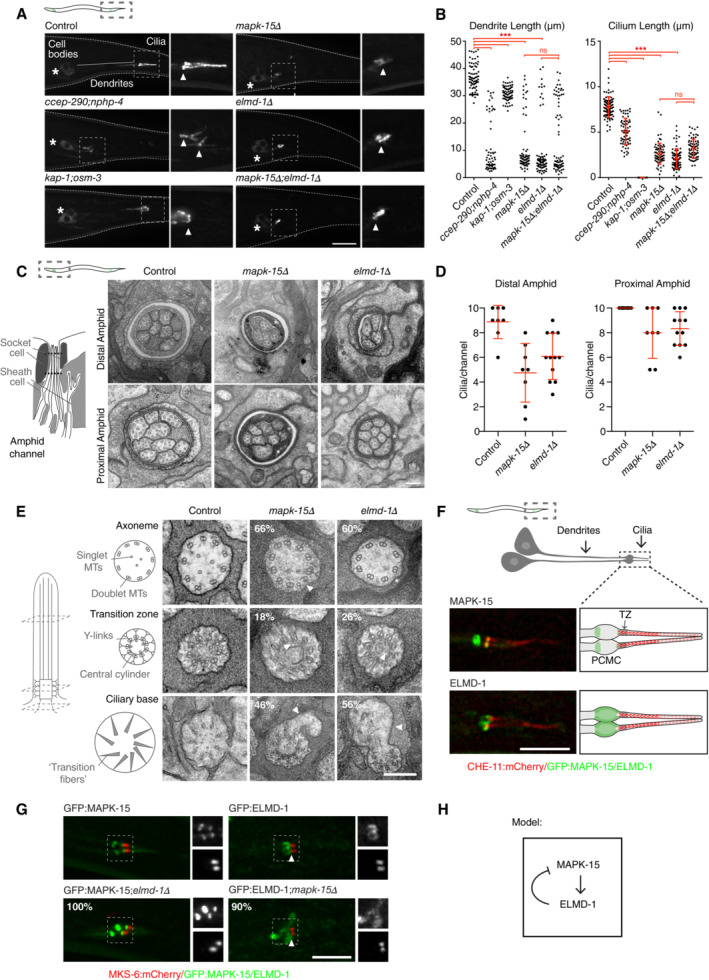
MAPK15 and ELMOD as highly conserved regulators of cilium biogenesis A, B
Full gene deletions of *mapk‐15* and *elmd‐1* in *C. elegans* result in both shortened cilia and shortened dendrites, combining the signature phenotypes of IFT and transition zone mutants (here *kap‐1;osm‐3* and *ccep‐290;nphp‐4* double mutants, respectively). Representative images of phasmid (tail) neurons expressing OSM‐6:GFP (A) and quantitation of cilium and dendrite lengths (B) in control and mutant animals as indicated. *n* > 20 animals for all conditions. Asterisks indicate statistically significant difference to wild type (cilium lengths, *t*‐test, ***P < 0.001, dendrite lengths, Wilcoxon Mann–Whitney test, ***P < 0.001). *mapk‐15;elmd‐1* double mutants do not display phenotypes stronger than either single mutant (*t*‐test and Wilcoxon Mann–Whitney test as above, not significant).C–E
*mapk‐15* and *elmd‐1* mutants display defects in ciliary ultrastructure. (C) Cross‐sectional views through the amphid channel reveal defects in cilium extension in both mutants, with fewer cilia protruding into the channel (asterisks). (D) Quantitation of cilium extension defects at the level of the proximal and distal amphid channel. (E) Higher magnification views of individual cilia reveal defects (indicated by arrowheads) at the level of the proximal segment of the axoneme (fewer than 9 doublet microtubules), transition zone (disorganization of the central cylinder and overall architecture), and ciliary base (membrane blebs). Percentages reported in figure based on examination of 4 *mapk‐15* and 7 *elmd‐1* mutant animals.F
Endogenous/Endogenous promoter GFP fusions to ELMD‐1 and MAPK‐15 localize to the ciliary base and periciliary membrane compartment in *C. elegans* phasmid neurons, the former marked by the IFT component CHE‐11.G, H
Localization interdependencies between MAPK‐15 and ELMD‐1. (G) Loss of ELMD‐1 results in a hyperaccumulation of MAPK‐15 at the *C. elegans* ciliary base, while loss of MAPK‐15 impairs localization of ELMD‐1, suggesting that (H) MAPK‐15 functions upstream of ELMD‐1. Percentages reported in figure based on examination of >30 animals for each condition. Data information: Scale bars are 10 μm (A), 200 nm (C, E), 100 nm (E, F), 5 μm (F, G), 1 μm. Error bars in (B, D) are standard deviation. Source data are available online for this figure.

**Figure EV1:**
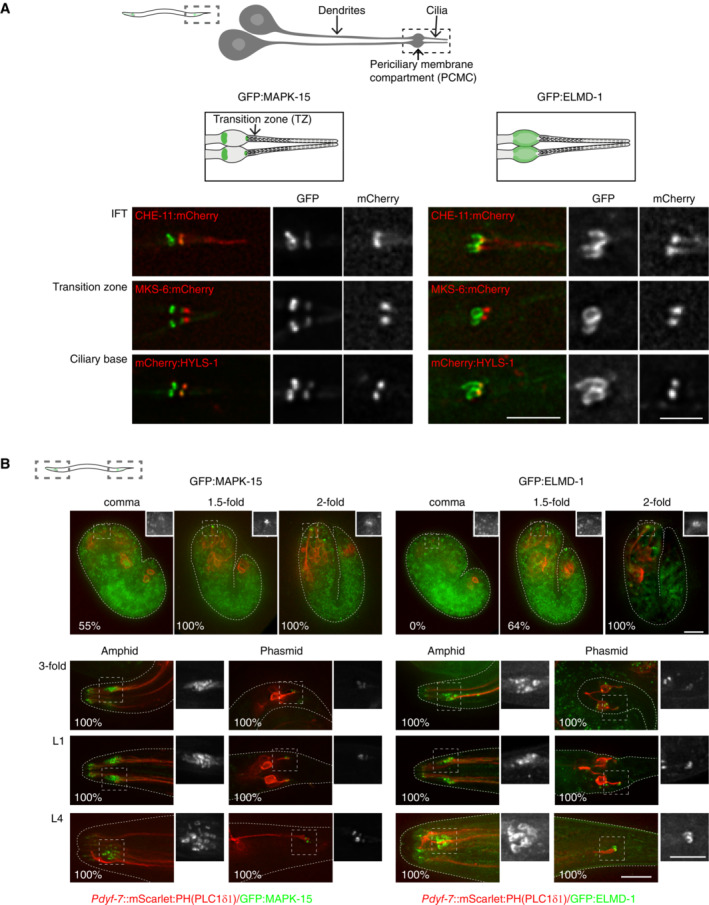
Further characterization of *C. elegans* MAPK‐15 and ELMD‐1 Detailed examination of MAPK‐15/ELMD‐1 localization in *C. elegans* phasmid neurons. Endogenous promoter GFP:MAPK‐15 co‐localizes with the basal body marker HYLS‐1 and accumulations of the IFT marker CHE‐11, just proximal to the transition zone marked by MKS‐6. A second population of MAPK‐15 localizes to the adhesion belt at the proximal end of the periciliary membrane compartment. Endogenously GFP‐tagged ELMD‐1 localizes to the entire periciliary membrane compartment.Analysis of MAPK‐15/ELMD‐1 recruitment during *C. elegans* embryogenesis. MAPK‐15 signal first becomes detectable in postmitotic sensory neurons marked by expression of a plasma membrane marker at the comma stage of embryogenesis (430 min after fertilization, Sulston *et al*, 1983), with ELMD‐1 signal following shortly thereafter at the 1.5‐fold stage (460 min), both proteins localizing to the distal tip of the elongating dendrite where the cilium will eventually form (Nechipurenko *et al*, 2017; Serwas *et al*, 2017). Neither GFP fusion is detectable at earlier stages of development or in other tissues of the worm. The hazy fluorescence signal in late stage embryos is due to autofluorescence. *n* > 10 animals for each developmental stage. Data information: Scale bars are 10 μm (A, B), 5 μm (A, B, insets).

**Figure EV2:**
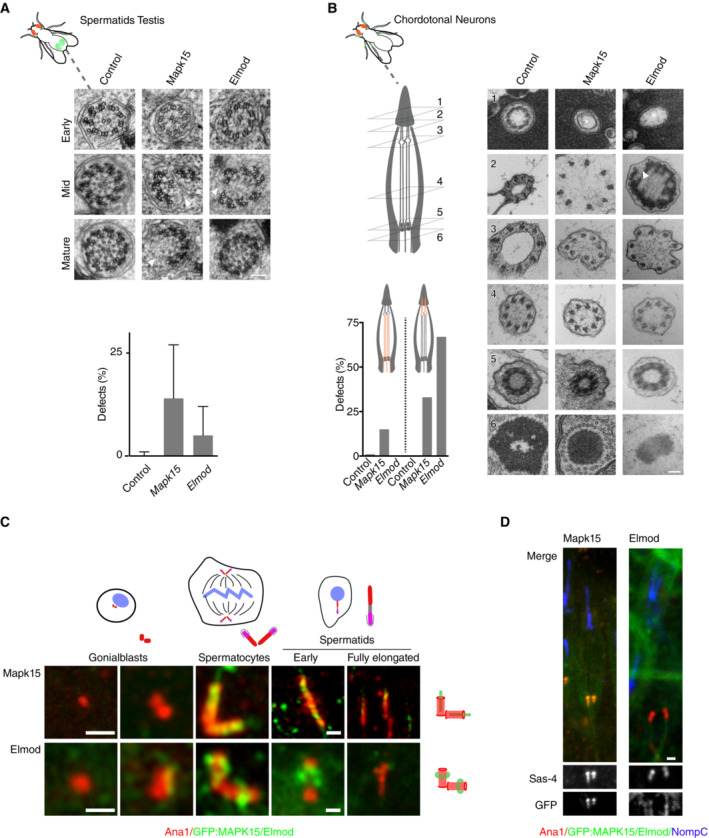
Characterization of Mapk15 and Elmod in *Drosophila* A, B
RNAi‐mediated depletion of MAPK15 and ELMOD in *Drosophila* sperm (A) and chordotonal neurons (B) results in defects in ciliary ultrastructure in both cellular contexts, with shortened (asterisks) and broken (arrowheads) axonemes in neurons and sperm, respectively. At least three animals examined per condition.C
GFP fusions to MAPK15 and ELMOD do not localize to centrioles marked by Ana1, but are recruited to maturing basal bodies during *Drosophila* spermatogenesis.D
MAPK15 and ELMOD co‐localize with the centriolar marker Sas‐4 on mature basal bodies in chordotonal neurons. Data information: Scale bars are 100 μm (A, B), 1 μm (C, D). Error bars in (A) are standard deviation. Source data are available online for this figure.

What caught our attention is that MAPK‐15 is one of only 15 genes universally conserved in ciliated species and lost in nonciliated ones, a remarkably short list that also includes the core centriolar structural components SASS6 and CENPJ/SAS‐4, suggesting a central role in cilium biogenesis (see Dataset EV2D). Consistent with this, MAPK15 scored strongly in the primary and secondary screens in the fly as well as in the worm. Given that available mutants in *C. elegans* are only partial gene deletions, a complete knockout was generated by CRISPR/Cas9‐mediated gene editing. This mutant presented an even stronger phenotype (with an average of 4.3 and 0.3 dye‐filled neurons in amphids and phasmids, respectively, compared to 6.9 and 0.4 for the original mutant), with cilium length and dendrite extension (indicative of transition zone dysfunction in the worm; Schouteden *et al*, 2015) both strongly reduced (Fig 7A and B). Ultrastructural analysis revealed that *mapk‐15* null mutants display severe defects in transition zone organization and axoneme elongation, with additional distortions of the periciliary membrane previously noted for loss of the microtubule organizing center at the ciliary base (Garbrecht *et al*, 2021; Fig 7C–E). This combination of phenotypes in the worm is remarkable in that IFT mutants do not affect transition zone organization, while loss of transition structures barely affects cilium elongation (Schouteden *et al*, 2015; Fig 7A and B). Indeed, no other previously characterized *C. elegans* gene shares this dual phenotype. The *Drosophila* phenotype is likewise remarkable: severe defects in both sperm and neuronal axoneme elongation and structural integrity (Fig EV2A and B). Defects in flagellar morphogenesis in particular are telling, given that this process is IFT‐independent in the fly. MAPK15 thus clearly functions in IFT‐independent step in axoneme elongation, consistent with its conservation in *Plasmodium*, which entirely lacks IFT components. MAPK15 has previously been reported to localize to the (acentriolar) ciliary base in *C. elegans* and basal bodies in vertebrates, as well as to the periciliary membrane compartment in *C. elegans* (Kazatskaya *et al*, 2017). We could confirm those observations for the worm (Figs 7F and EV1A) and extend them to *Drosophila*, where MAPK15 co‐localized with the core centriolar component Sas4 to basal bodies in both chordotonal neurons and sperm (Fig EV2C and D). What is worth noting, however, is that MAPK15 does not localize to centrioles elsewhere in the worm or fly. Instead, MAPK15 is recruited to basal bodies at a time comparable to the expression of the very first ciliary components (1.5‐fold stage in *C. elegans* embryogenesis; Serwas *et al*, 2017; Fig EV1B), meiosis I in *Drosophila* spermatogenesis (Basiri *et al*, 2014; Fig EV2C). In summary, then, what MAPK15 represents is a universally conserved regulator that is recruited at the onset of ciliogenesis to initiate the assembly of otherwise largely independent ciliary structures (the ciliary transition zone and axoneme).

ELMOD1‐3, represented in *Drosophila* and *C. elegans* by a single ortholog, ELMOD/ELMD‐1, are part of a family of ELMO (Engulfment and cell motility) domain‐containing proteins, named after its founding member CED‐12/ELMO1, which acts to promote apoptotic cell engulfment by activating CED‐10/Rac1 GTPase (Wu *et al*, 2001; Zhou *et al*, 2001). In contrast, ELMOD family members are thought to act as ARF/ARL GAPs, with broad cellular effects, including on the actin cytoskeleton (Li *et al*, 2019b). All three vertebrate ELMOD proteins have recently been localized to basal bodies and linked to ciliogenesis, albeit with seemingly opposing effects: While loss of ELMOD1 and 3 reduced ciliation in mouse embryonic fibroblasts, loss of ELMOD2 actually resulted in an increase, with those cilia displaying abnormal morphology, including apparent splaying of axonemes and ciliary rootlet defects (Turn *et al*, 2021, 2022; ultrastructural analysis has so far not been performed). Common to all three perturbations is a mislocalization of ciliary proteins, suggesting ciliary trafficking is at least partly responsible for the observed defects (Turn *et al*, 2021, 2022). In our primary and secondary screen in *Drosophila*, ELMOD presented with strong phenotypes in both sperm and neurons and rather less of a phenotype in *C. elegans*. However, the latter result was based on a nonsense mutant of uncertain consequence (*elmd‐1(gk386113)*, Trp24Ter; Thompson *et al*, 2013). Similarly, weak phenotypes were reported recently for another mutant, *elmd‐1(syb630)*, a partial gene deletion (preprint: Cevik *et al*, 2021). We therefore obtained a full gene deletion, which yielded a much stronger phenotype (with an average of 4.9 and 0.5 dye‐filled neurons in amphids and phasmids, respectively, compared to 10.4 and 3.8 for the original mutant) and was used for further analysis. Remarkably, in both *C. elegans* and *Drosophila* ELMOD presented phenotypes nearly indistinguishable from MAPK15. Thus, in *C. elegans* both cilium and dendrite lengths were much reduced, with axonemal, transition zone, and periciliary membrane compartment defects matching those of MAPK15 (Fig 7A–E). Likewise, in *Drosophila* ELMOD depletion led to defects in axoneme elongation and structural integrity in both sperm and chordotonal neurons (Fig EV2A and B). ELMOD and MAPK15 localization was also similar if not quite identical: In *Drosophila*, both proteins localize to basal bodies (Fig EV2C and D), while in *C. elegans* both display a dual localization to the ciliary base as well as the periciliary membrane compartment, although here the domain occupied by ELMOD is more extensive (Figs 7F and EV1A). Finally, neither protein localizes to centrioles, with both being recruited to basal bodies early in ciliogenesis with near‐identical timing in *Drosophila* sperm and *C. elegans* neurons (Figs EV1B and EV2C). These similarities led us to posit that these proteins may function in the same molecular pathway. Consistent with this, *elmd‐1;mapk‐15* double mutants in *C. elegans* presented a phenotype no more severe than that of either single mutant (Fig 7A and B). Finally, we sought to investigate whether there were any localization interdependencies between these two ciliary components. We found loss of MAPK‐15 to impair proper ciliary targeting of ELMD‐1, while MAPK‐15 localization was not affected by loss of ELMD‐1 (indeed, levels were markedly increased; Fig 7G). Our results therefore indicate that ELMD‐1 functions downstream of MAPK‐15 in a ciliogenesis program that serves to convert centrosome‐organizing centrioles into basal bodies capable of supporting axoneme extension and transition zone assembly (Fig 7H).

## Discussion

Cilia in many ways are ideally suited for comparative genomics approaches in that they are evolutionarily ancient yet not universally conserved in all extant species. Once cilia are no longer present, gene loss follows very rapidly, as demonstrated by the example of *Emiliania huxleyi*, where haploid‐specific genes including those required for cilium biogenesis have been found to have been lost multiple times in different subpopulations of the same species (von Dassow *et al*, 2015). The resultant presence and absence pattern for ciliary genes in the genomes of fully sequenced eukaryotes creates a prominent phylogenetic signature that sets them apart from other highly conserved genes. We found that, at least for cilia, a simple but robust phylogenetic profiling approach based on bidirectional best matches and hierarchical clustering outperforms more sophisticated approaches and identifies a single cluster of 386 genes. This cluster includes the vast majority of known players involved in key steps of centriole assembly, cilium biogenesis, and motility, as well as a set of novel genes that our functional analysis indicates are likewise associated with different aspects of cilium assembly and motility. We believe this set to constitute the core complement of genes required to assemble motile cilia. The phylogenetic distribution of these 386 genes indicates that all of them originate in the last eukaryotic common ancestor. In contrast to the rather simple molecular architecture of centrioles (Pelletier *et al*, 2006), the larger basal body‐cilium superstructure of which they form an integral part then is highly complex and has been for > 1,000 million years.

Importantly, we have carried out a comprehensive verification of our bioinformatic predictions by performing a systematic RNAi‐based analysis in *Drosophila* as well as a more targeted mutant analysis in *C. elegans* of the genes within our cluster to examine the phenotypic consequences of their perturbation. In both experimental models, novel candidate genes scored at a similar frequency to known genes within the cluster, without presenting any significant pleiotropic phenotypes. Given the limitations in sensitivity of the assays employed in our screen, we conclude that most if not all candidate genes are indeed cilium‐linked. We were unable to assess this for the 64 genes not conserved in either worm or fly. However, the plentiful indications of cilium‐related functions from large‐scale proteomic, expression, and localization approaches give us the confidence to extend this conclusion to the entire set of 152 genes within our cluster. The use of *C. elegans* and *Drosophila* as complementary experimental models enabled us to classify novel genes into genes required for cilium biogenesis (the only class conserved in *C. elegans*) or cilium motility, with the former category further subdivided into genes required specifically for compartmentalized cilium biogenesis (scoring in *Drosophila* neurons only) and those required for cilium biogenesis regardless of context (scoring in both *Drosophila* neurons and sperm).

In our downstream characterization, we focused on genes required for cilium biogenesis, particularly those involved in the poorly understood early steps surrounding the conversion of centrioles into basal bodies and initiation of transition zone/axoneme assembly. Given the almost total lack of known biogenesis genes conserved in *Plasmodium* where this process must of necessity occur in an IFT‐independent manner, we were also interested in any genes required for IFT‐independent cilium biogenesis in *Drosophila* sperm. The first set of genes comprises the novel basal body components CABLES1, CCDC6, and TEX9. We found these genes to function in a pathway initiated by the cartwheel component Bld10/CEP135 and also involving the microtubule plus‐end tracking protein CEP104. CEP135 is notable for being part of a highly conserved centriole and basal body module first identified by the laboratory of Monica Bettencourt‐Dias, also comprising SASS6 and SAS‐4/CENPJ (Carvalho‐Santos *et al*, 2010). Yet, unlike SASS6 and SAS‐4/CENPJ, CEP135 is not required for centriole assembly itself, instead playing a role specifically in the formation of basal bodies (Roque *et al*, 2012). We see CABLES1, CCDC6, and TEX9 as part of the same centriole to basal body conversion program. While highly conserved, not all components of this program are present in every ciliated species (by our criteria only CABLES1 and CEP104 are universally conserved, including in *Plasmodium*), nor are they necessarily required in every cellular context within the same organism. We suggest that this reflects an underappreciated variability in basal body and ciliary architecture in different species and different tissues within the same species, building on a common core centriole structure (Jana *et al*, 2018; Jana, 2021). A different form of diversification is exemplified by the CFAP97 and CFAP299 family of genes, where multiple paralogs exist in *Drosophila* and vertebrates. It is notable that different family members in many cases have different tissue expression patterns, raising the possibility of both redundancy and functional diversification. Further work will be required to establish the degree to which paralogs can functionally replace each other when expressed in the respective mutant context. When considered as a family, both CFAP97 and CFAP299 function in core (i.e., IFT‐independent) cilium biogenesis, with phenotypes in both *Drosophila* sperm and neurons, although only CFAP299 is conserved in *Plasmodium*. As described above, CFAP299 presents a particularly unusual phenotype in sperm, with a failure of membrane deposition around the flagellar axoneme. How this phenotype relates to its function in canonical, compartmentalized cilium biogenesis remains to be established, but it is clear that this is a particularly interesting candidate to follow up on.

Finally, the atypical MAP kinase MAPK15 is of interest as a universally conserved master regulator of ciliogenesis, present in all ciliated species and lost in nonciliated ones. Such a high degree of conservation stands in contrast to the regulation of centriole assembly, which only in metazoans is driven by the polo‐like kinase PLK4/ZYG‐1, while core centriolar structural components are universal across eukaryotes (Carvalho‐Santos *et al*, 2010). At the same time, the loss of MAPK15 in nonciliated species argues against pleiotropic functions such as have been proposed for this kinase in vertebrates (Deniz *et al*, 2022). These observations speak to a high degree of evolutionary constraint, potentially reflecting multiple targets within the ciliogenesis pathway. We here identify one downstream effector, the putative ARF/ARL GAP ELMOD, which is likewise highly (but not universally) conserved and which displays a near‐identical spectrum of phenotypes to MAPK15 in *C. elegans* and *Drosophila*. The downstream targets for MAPK15 in ciliogenesis beyond ELMOD are currently unclear. While previous work has linked both MAPK15 and ELMOD to ciliary trafficking (Kazatskaya *et al*, 2017; preprint: Cevik *et al*, 2021; Turn *et al*, 2021, 2022), this clearly cannot explain their role in IFT‐independent cilium biogenesis in *Drosophila* sperm and (in the case of MAPK15) *Plasmodium*. It is also notable that no other *C. elegans* gene to date shares their dual phenotype in transition zone assembly and axoneme extension. With both proteins being recruited at the earliest stages of ciliogenesis as centrioles become basal bodies, we suggest that their role is instead in that initial templating step, similar to the role ascribed to TTBK2, a kinase not clearly linked to ciliogenesis beyond metazoans, in the intracellular pathway of ciliogenesis (Goetz *et al*, 2012).

In conclusion, our phylogenetic profiling‐based screen has defined what appears to be the core set of genes required for cilium assembly and motility across eukaryotes, a set that includes a substantial number of as yet poorly characterized candidate genes. With many ciliary cluster genes linked to ciliopathies, we believe this dataset to represent an invaluable resource for basic researchers and clinicians alike.

## Materials and Methods

### Reagents and Tools table

**Table:** 

Reagent/Resource	Reference or Source	Identifier or Catalog Number
**Experimental models**		
*C. elegans*: Strain N2: *C. elegans wild type (ancestral)*	CGC	N2
*C. elegans*: Strains for primary screen detailed in Dataset EV3A	As indicated in table	
*C. elegans*: Strain DAM839: *vuaSi15 [pBP36; Posm‐6::osm‐6::eGFP; cb‐unc‐119(+)] I; vuaSi21 [pBP39; Pmks‐6::mks‐6::mCherry; cb‐unc‐119(+)] II; unc‐119(ed3) III; osm‐6(p811) V*	(Serwas *et al*, 2017)	DAM839
*C. elegans*: Strain DAM976: *mapk‐15(vie30[pAD696; mapk‐15::loxP])III*	This study	DAM976
*C. elegans*: Strain DAM1012: *vuaSi15 [pBP36; Posm‐6::osm‐6::eGFP; cb‐unc‐119(+)] I; vuaSi21 [pBP39; Pmks‐6::mks‐6::mCherry; cb‐unc‐119(+)] II; mapk‐15(vie30[pAD696; C05D10.2::loxP])III; osm‐6(p811) V*	This study	DAM1012
*C. elegans*: Strain DAM1046: *mapk‐15(vie30[pAD696; mapk‐15::loxP])III; vieSi103[pAD690; Pmapk‐15::GFP::mapk‐15; cb‐unc‐119(+)] IV; vieSi16[pAD390; Phyls1:mcherry::hyls‐1; cb‐unc‐119(+)]IV*	This study	DAM1046
*C. elegans*: Strain DAM1085: *elmd‐1(syb508) III (6x outcrossed)*	This study	DAM1085
*C. elegans*: Strain DAM1088: *vuaSi15 [pBP36; Posm‐6::osm‐6::eGFP; cb‐unc‐119(+)] I; vuaSi21 [pBP39; Pmks‐6::mks‐6::mCherry; cb‐unc‐119(+)] II; elmd‐1(syb508) III; osm‐6(p811) V*	This study	DAM1088
*C. elegans*: Strain DAM1137: *elmd‐1(syb1113) III* (4x outcrossed)	This study	DAM1137
*C. elegans*: Strain DAM1141: *elmd‐1(syb1113) III; vieSi16[pAD390; Phyls1:mcherry::hyls‐1; cb‐unc‐119(+)]IV*	This study	DAM1141
*C. elegans*: Strain DAM1152: *vuaSi21 [pBP39; Pmks‐6::mks‐6::mCherry; cb‐unc‐119(+)] II; elmd‐1(syb1113) III; mapk‐15(vie30[pAD696; mapk‐15::loxP])III*	This study	DAM1152
*C. elegans*: Strain DAM1153: *vuaSi21 [pBP39; Pmks‐6::mks‐6::mCherry; cb‐unc‐119(+)] II; elmd‐1(syb508) III; vieSi103[pAD690; Pmapk‐15::GFP::mapk‐15; cb‐unc‐119(+)] IV*	This study	DAM1153
*C. elegans*: Strain DAM1154: *vuaSi21 [pBP39; Pmks‐6::mks‐6::mCherry; cb‐unc‐119(+)] II; elmd‐1(syb1113) III*	This study	DAM1154
*C. elegans*: Strain DAM1157: *vuaSi21 [pBP39; Pmks‐6::mks‐6::mCherry; cb‐unc‐119(+)] II; mapk‐15(vie30[pAD696; mapk‐15::loxP])III; vieSi103[pAD690; Pmapk‐15::GFP::mapk‐15; cb‐unc‐119(+)] IV*	This study	DAM1157
*C. elegans*: Strain DAM1169: *vuaSi15 [pBP36; Posm‐6::osm‐6::eGFP; cb‐unc‐119(+)] I; vuaSi21 [pBP39; Pmks‐6::mks‐6::mCherry; cb‐unc‐119(+)] II; mapk‐15(vie30[pAD696; mapk‐15::loxP])III; elmd‐1(syb508) III*	This study	DAM1169
*C. elegans*: Strain DAM1176: *mapk‐15(vie30[pAD696; mapk‐15::loxP])III; vieSi103[pAD690; Pmapk‐15::GFP::mapk‐15; cb‐unc‐119(+)] IV; vieSi124[pAD791; pDC611 Pdyf‐7‐mScarlet‐PH‐unc‐54‐3’UTR; cb‐unc‐119(+)]V*	This study	DAM1176
*C. elegans*: Strain DAM1180: *elmd‐1(syb1113) III; vieSi124[pAD791; pDC611 Pdyf‐7‐mScarlet‐PH‐unc‐54‐3’UTR; cb‐unc‐119(+)]V*	This study	DAM1180
*C. elegans*: Strain DAM1331: *ccep‐290(tm4927)I; vuaSi15 [pBP36; Posm‐6::osm‐6::eGFP; cb‐unc‐119(+)] I; vuaSi21 [pBP39; Pmks‐6::mks‐6::mCherry; cb‐unc‐119(+)] II; unc‐119(ed3) III; nphp‐4(tm925)V; osm‐6(p811) V*	This study	DAM1331
*C. elegans*: Strain DAM1332: *vuaSi15 [pBP36; Posm‐6::osm‐6::eGFP; cb‐unc‐119(+)] I; kap‐1(ok676) II; vuaSi21 [pBP39; Pmks‐6::mks‐6::mCherry; cb‐unc‐119(+)] II; unc‐119(ed3) III; osm‐3(p802)IV; osm‐6(p811) V*	This study	DAM1332
*C. elegans*: Strain DAM1338: *vuaSi24 [pBP43; Pche‐11::che‐11::mCherry; cb‐unc‐119(+)] II; mapk‐15(vie30[pAD696; mapk‐15::loxP])III; vieSi103[pAD690; Pmapk‐15::GFP::mapk‐15; cb‐unc‐119(+)] IV; che‐11(tm3433) V?*	This study	DAM1338
*C. elegans*: Strain DAM1339: *vuaSi24 [pBP43; Pche‐11::che‐11::mCherry; cb‐unc‐119(+)] II; elmd‐1(syb1113) III; che‐11(tm3433) V*	This study	DAM1339
*C. elegans*: Strain DAM1346: *vuaSi21 [pBP39; Pmks‐6::mks‐6::mCherry; cb‐unc‐119(+)] II; vieSi103[pAD690; Pmapk‐15::GFP::mapk‐15; cb‐unc‐119(+)] IV*	This study	DAM1346
*C. elegans*: Strain PHX508: *elmd‐1(syb508) III*	SunyBiotech	PHX508
*C. elegans*: Strain PHX1113: *elmd‐1(syb1113) III*	SunyBiotech	PHX1113
*D. melanogaster*: Wild type: w[1118]	BDSC	RRID:BDSC_6326
*D. melanogaster*: Bam‐GAL4 & Nanos‐GAL4: P{GAL4‐nos.NGT}40; P{bam‐GAL4:VP16}	Helen White‐Cooper; BDSC	N/A; RRID:BDSC_4442
*D. melanogaster*: Elav‐GAL4: P{w[+mW.hs] = GawB}elav[C155] w[1118];; P{w[+mC] = UAS‐Dcr‐2.D}10	BDSC	RRID:BDSC_458
*D. melanogaster*: Tub‐GAL4: P{w[+mC] = tubP‐GAL4}LL7/TM6, Tb Sb	BDSC	RRID:BDSC_5138
*D. melanogaster*: GD RNAi fly stocks: w1118; P{GD.CloneID}v10005	VDRC	RNAi construct ID (see Dataset EV3)
*D. melanogaster*: KK RNAi fly stocks: y,w[1118];P{KK.CloneID}VIE‐260B	VDRC	RNAi construct ID (see Dataset EV3)
*D. melanogaster*: TRiP RNAi fly stocks: y[1] v[1]; P{y[+t7.7] v[+t1.8] = TRiP.CloneID}attP40 (2nd chromosome); or: y[1] v[1]; P{y[+t7.7] v[+t1.8] = TRiP.CloneID}attP2 (3rd chromosome)	BDSC	RNAi construct ID (see Dataset EV3)
*D. melanogaster*: Cables1Δ	This study	N/A
*D. melanogaster*: Cep104Δ	This study	N/A
*D. melanogaster*: Cep135Δ	Singh *et al* (2014)	N/A
*D. melanogaster*: CFAP299Δ	This study	N/A
*D. melanogaster*: CCDC6‐GFP	This study	N/A
*D. melanogaster*: Cep104‐GFP	This study	N/A
*D. melanogaster*: GFP‐Cables1	This study	N/A
*D. melanogaster*: GFP‐CEP135	Roque *et al* (2012)	N/A
*D. melanogaster*: GFP‐CFAP97D1	This study	N/A
*D. melanogaster*: GFP‐Elmod	This study	N/A
*D. melanogaster*: GFP‐MAPK15	This study	N/A
*D. melanogaster*: Tex9‐GFP	Sarov *et al* (2016)	VDRC #318643
**Recombinant DNA**		
*C. elegans*: CRISPR repair template for ctg‐1Δ (500 bp flanking sequences) in pUC57 vector	This study	pAD718
*C. elegans*: CRISPR repair template for mapk‐15Δ (500 bp flanking sequences) in pUC57 vector	This study	pAD696
*C. elegans*: CRISPR repair template for pgam‐5Δ (500 bp flanking sequences) in pUC57 vector	This study	pAD703
*C. elegans*: CRISPR repair template for tag‐321Δ (500 bp flanking sequences) in pUC57 vector	This study	pAD716
*C. elegans*: Mos Co‐injection marker pCFJ104‐Pmyo‐3::mCherry::unc‐54	Frokjaer‐Jensen *et al* (2008)	Addgene Plasmid # 19328
*C. elegans*: Mos Co‐injection marker pCFJ90‐Pmyo‐2::mCherry::unc‐54utr	Frokjaer‐Jensen *et al* (2008)	Addgene Plasmid # 19327
*C. elegans*: Mos Co‐injection marker pGH8‐pRAB‐3::mCherry::unc‐54utr	Frokjaer‐Jensen *et al* (2008)	Addgene Plasmid # 19359
*C. elegans*: Mos vector pCFJ151	Frokjaer‐Jensen *et al* (2008)	Addgene Plasmid # 19330
*D. melanogaster*: CRISPR gRNA expression vector pCFD4	Port *et al* (2014)	Addgene Plasmid # 49411
*D. melanogaster*: CRISPR gRNA expression vector pCFD4 CFAP299	This study	N/A
*D. melanogaster*: Vector pDsRed‐attP	Gratz *et al* (2015)	Addgene Plasmid # 51019
*D. melanogaster*: Vector pDsRed‐CRISPR CFAP299	This study	N/A
*D. melanogaster*: Gateway vector pDONR Zeo	Thermo Fisher	Cat# 12535035
*D. melanogaster*: Gateway vector pUbq‐GFPNT	Stevens *et al* (2010)	N/A
*D. melanogaster*: Gateway vector pUbq‐GFPCT	Stevens *et al* (2010)	N/A
*D. melanogaster*: Vector pUbq‐GFP‐Cables1	This study	N/A
*D. melanogaster*: Vector pUbq‐CCDC6‐GFP	This study	N/A
*D. melanogaster*: Vector pUbq‐Cep104‐GFP	This study	N/A
*D. melanogaster*: Vector pUbq‐GFP‐CFAP97D1	This study	N/A
*D. melanogaster*: Vector pUbq‐GFP‐Elmod	This study	N/A
*D. melanogaster*: Vector pUbq‐GFP‐MAPK15	This study	N/A
**Antibodies**		
Alpaca GFP‐Booster Atto 488‐conjugated nanobody	ChromoTek	Cat#gba488‐100; RRID: AB_2631386
*D. melanogaster*: Mouse monoclonal anti‐gamma Tubulin antibody	Sigma‐Aldrich	Cat# T6557, RRID:AB_477584
*D. melanogaster*: Mouse monoclonal anti‐NompC antibody	Liang *et al* (2011)	N/A
*D. melanogaster*: Rabbit polyclonal anti‐Ana1 antibody	Conduit *et al* (2010)	N/A
*D. melanogaster*: Rabbit polyclonal anti‐Cep135 antibody	Roque *et al* (2012)	N/A
*D. melanogaster*: Rabbit polyclonal anti‐Sas4 antibody	Basto *et al* (2006)	N/A
Donkey anti‐Rabbit IgG (H + L) Alexa Fluor 488‐conjugated antibody	Thermo Fisher	Cat#A‐21206; RRID: AB_2535792
Goat anti‐Mouse IgG (H + L) Alexa Fluor 568‐conjugated antibody	Thermo Fisher	Cat#A‐11004; RRID: AB_2534072
Goat anti‐Mouse IgG (H + L) Alexa Fluor 647‐conjugated antibody	Thermo Fisher	Cat#A‐21236; RRID: AB_2535805 conjugated antibody
Goat anti‐Rabbit IgG (H + L) Alexa Fluor 568‐conjugated antibody	Thermo Fisher	Cat#A‐11011; RRID: AB_143157
**Oligonucleotides and other sequence‐based reagents**		
*C. elegans*: *ctg‐1* CRISPR crRNA 1 (target sequence: GTTCATTGCATCGAGTAATGT)	This study	N/A
*C. elegans*: *ctg‐1* CRISPR crRNA 2 (target sequence: GAATATTGTAGCGCAATTGCT)	This study	N/A
*C. elegans*: *dpy‐10* co‐CRISPR crRNA (target sequence: GCUACCAUAGGCACCACGAG)	Paix *et al* (2015)	N/A
*C. elegans*: *dpy‐10* co‐CRISPR repair template: CACTTGAACTTCAATACGGCAAGATGAGAATGACTGGAAACCGTACCGCATGCGGTGCCTATGGTAGCGGAGCTT CACATGGCTTCAGACCAACAGCCTAT	Paix *et al* (2015)	N/A
*C. elegans*: *mapk‐15* CRISPR crRNA 1 (target sequence: AATGTGCGTGTCCACATCGT)	This study	N/A
*C. elegans*: *mapk‐15* CRISPR crRNA 2 (target sequence: TTTGTGTGGGTGAAGGATCT)	This study	N/A
*C. elegans*: *pgam‐5* CRISPR crRNA 1 (target sequence: GTATCTAAGATCATAAAACT)	This study	N/A
*C. elegans*: *pgam‐5* CRISPR crRNA 2 (target sequence: AGGAAATCTTGTTTGGTGGA)	This study	N/A
*C. elegans*: *tag‐321* CRISPR crRNA 1 (target sequence: GTCGTCGCTGAAGAAAAGTAA)	This study	N/A
*C. elegans*: *tag‐321* CRISPR crRNA 2 (target sequence: GATCAGCTCGCCAGCTCGTC)	This study	N/A
*C. elegans*: tracrRNA: AACAGCAUAGCAAGUUAAAAUAAGGCUAGUCCGUUAUCAACUUGAAAAAGUGGCACCGAGUCGGUGCUUUUUUU	Dharmacon	Cat# U‐002005‐200
*D. melanogaster*: pCFD4 CFAP299 f: TATATAGGAAAGATATCCGGGTGAACTTCGCACTCCTTTACGACCGTCGGTTTAGAGCTAGAAATAGCAAG	This study	N/A
*D. melanogaster*: pCFD4 CFAP299 r: ATTTTAACTTGCTATTTCTAGCTCTAAAACTGTTCCGGATGAGCTACCACGACGTTAAATTGAAAATAGGTC	This study	N/A
*D. melanogaster*: pDsRed‐CRISPR CFAP299 1f: GGAACACCTGCGATCTCGCGAGCCTCTCATTGATAATTC	This study	N/A
*D. melanogaster*: pDsRed‐CRISPR CFAP299 1r: GGAACACCTGCTTCACTACGATGAGCAAATATTTTAGCAC	This study	N/A
*D. melanogaster*: pDsRed‐CRISPR CFAP299 2f: GGAAGCTCTTCATATATTATGTTCAACTAATGTTTTG	This study	N/A
*D. melanogaster*: pDsRed‐CRISPR CFAP299 2r: GGAAGCYCTTCAGACCTTTCACAATAGACTGGTCAC	This study	N/A
*D. melanogaster*: GFP‐Cables1 f: GGGGACAAGTTTGTACAAAAAAGCAGGCTTGATGGCAAGTTCGCTCAACAAG	This study	N/A
*D. melanogaster*: GFP‐Cables1 r: GGGGACCACTTTGTACAAGAAAGCTGGGTGCTAGGACTCGTAGAGCAGCCGCTG	This study	N/A
*D. melanogaster*: CCDC6‐GFP f: GGGGACAAGTTTGTACAAAAAAGCAGGCTTGATGGAAAGTCCCTGTGAATC	This study	N/A
*D. melanogaster*: CCDC6‐GFP r: GGGGACCACTTTGTACAAGAAAGCTGGGTGGACACGTCCACCTGCAAGGAC	This study	N/A
*D. melanogaster*: Cep104‐GFP f: GGGGACAAGTTTGTACAAAAAAGCAGGCTTGATGGCTAAGAAAATACCTTTTAACG	This study	N/A
*D. melanogaster*: Cep104‐GFP r: GGGGACCACTTTGTACAAGAAAGCTGGGTGATTTGACTTCTTGAGATTC	This study	N/A
*D. melanogaster*: GFP‐CFAP97D1 f: GGGGACAAGTTTGTACAAAAAAGCAGGCTTGATGATCTCACGACGCGACAAATTG	This study	N/A
*D. melanogaster*: GFP‐CFAP97D1 r: GGGGACCACTTTGTACAAGAAAGCTGGGTGTCATTCGTCGTAGGAGTAAGAGTAG	This study	N/A
*D. melanogaster*: GFP‐Elmod f: GGGGACAAGTTTGTACAAAAAAGCAGGCTTGATGTTTATTCTCGACCGAATAC	This study	N/A
*D. melanogaster*: GFP‐Elmod r: GGGGACCACTTTGTACAAGAAAGCTGGGTGTTAGACGGATTCTACAACCAAATTG	This study	N/A
*D. melanogaster*: GFP‐MAPK15 f: GGGGACAAGTTTGTACAAAAAAGCAGGCTTGATGGCCAACTATCAAACTGCCCAC	This study	N/A
*D. melanogaster*: GFP‐MAPK15 r: GGGGACCACTTTGTACAAGAAAGCTGGGTGTCAGTTGCTCTCCGGCAAGGAG	This study	N/A
**Chemicals, Enzymes and other reagents**		
Agar 100 Resin	Agar Scientific	Cat# AGR1031; CAS: 25038–04‐4
DiI	Invitrogen	Cat# V22885
Glutaraldehyde 25% EM grade	Agar Scientific	Cat# AGR1020; CAS: 111–30‐8
Hoechst 33258	Thermo Fisher	Cat# H1398; CAS: 23491–45‐4
Osmium Tetroxide 4% Solution	Electron Microscopy Sciences	Cat# 19170; CAS: 20816–12‐0
Phalloidin Alexa Fluor 568	Thermo Fisher	Cat# A12380
Propyl gallate	Sigma‐Aldrich	Cat# P3130; CAS: 121–79‐9
Reynolds lead citrate	Delta Microscopies	Cat# 11300; CAS: 512–26‐5
Tetramisole hydrochloride	Sigma‐Aldrich	Cat# L9756; CAS: 16595–80‐5
Uranyl acetate	Science Services	Cat# E22400; CAS: 541–09‐3
**Software**		
Cluster 3.0	de Hoon *et al* (2004)	http://bonsai.hgc.jp/~mdehoon/software/cluster/
Fiji v 2.0.0	NIH	https://imagej.net/software/fiji/
GraphPad Prism 6	GraphPad	https://www.graphpad.com/scientific-software/prism/
Jalview v 2.11.2.5	Waterhouse *et al* (2009)	http://www.jalview.org
Java TreeView v 1.1.6r4	Saldanha (2004)	http://jtreeview.sourceforge.net/
NCBI BLAST 2.2.28	Altschul *et al* (1997)	ftp://ftp.ncbi.nlm.nih.gov/blast/executables/blast+/LATEST/

### Methods and Protocols

#### Experimental model and subject details

##### *C. elegans* strains and culture conditions

All strains used in this study are listed in Dataset EV3A (primary dye‐fill screen) and Reagents and Tools Table (further analysis). Mutant strains for the primary screen were obtained from the National BioResource Project (NBRP, *tm* alleles not available at the CGC) or *Caenorhabditis* Genetics Center (CGC, all others). Detailed information on all mutants is available on WormBase (https://www.wormbase.org). Strains expressing endogenous promoter‐driven CHE‐11:mCherry (Prevo *et al*, 2015), mCherry:HYLS‐1 (Schouteden *et al*, 2015), MKS‐6:mCherry (Prevo *et al*, 2015), and OSM‐6:GFP (Prevo *et al*, 2015), and the loss of function alleles *ccep‐290(tm4927)*, *nphp‐4(tm925)* (Schouteden *et al*, 2015), *kap‐1(ok676)*, and *osm‐3(p802*) (Snow *et al*, 2004) have been previously described. Full gene deletions for *mapk‐15*, *ctg‐1*, *pgam‐5*, and *tag‐321* were generated by CRISPR/Cas9‐mediated homologous recombination using a plasmid‐based protocol (Dickinson *et al*, 2013). Successful deletion was detected using single worm PCR and confirmed by PCR and sequencing. A full gene deletion for *C56G7.3/elmd‐1* was generated by SunyBiotech and verified by PCR and sequencing. A strain expressing mScarlet:PH in neurons was generated by cloning the PH domain of rat PLC1δ1 (Kachur *et al*, 2008) under the control of the pDyf‐7 promoter and unc‐54 3’regulatory sequences with an N‐terminal mScarlet into the pCFJ151 targeting vector and Mos1‐mediated transposition (Frokjaer‐Jensen *et al*, 2008). A strain expressing endogenous promoter‐driven GFP:MAPK‐15 was similarly generated by cloning the genomic locus plus 5′ and 3′ regulatory sequences with an N‐terminal GFP into pCFJ151 and Mos1‐mediated transposition. Finally, a strain expressing endogenously GFP‐tagged ELMD‐1 was generated by SunyBiotech. Dual color strains and strains carrying mutant alleles were constructed by mating. *mapk‐15* and *elmd‐1* mutants were outcrossed multiple times against wild type prior to analysis/introduction of fluorescent markers. All strains were maintained at 20–23°C.

##### *Drosophila melanogaster* stocks and husbandry

All strains used in this study are listed in Dataset EV3A (primary and secondary screens) and Reagents and Tools Table (further analysis). Strains were obtained from Helen White‐Cooper (Bam‐GAL4 driver line), the Bloomington *Drosophila* Stock Center (BDSC, w[1118] allele, Tub‐GAL4, Elav‐GAL4, and Nanos‐GAL4 driver lines, as well as TRiP RNAi fly stocks) and Vienna *Drosophila* Resource Center (VDRC, GD, and KK RNAi fly stocks). Detailed information for these strains is available on FlyBase (http://flybase.org), as well as on the websites of the two stock centers (BDSC, https://bdsc.indiana.edu, VDRC, https://stockcenter.vdrc.at/control/main). Cep135 mutants were obtained from the Cabernard laboratory. Cep104 and Cables1 mutants were generated by Flp‐FRT recombination between two transposon elements flanking its coding sequence (Parks *et al*, 2004). In the case of Cep104 (CG10137), these were PBac{RB}CG10137e00472 and PBac{WH}CG10137f04546, in the case of Cables1 (CG6191) PBac{RB}CG6191e03779 and P{XP}CG6191d03529. To obtain complete deletions, males carrying one element were mated with females carrying a FLP recombinase transgene. Progeny males carrying both the element and FLP recombinase were then mated to females carrying the other element. After 2–3 days, parents were transferred to a new vial and progeny subjected to a 1 h heat shock by placing the vials into a 37°C water bath. We subjected the vials to daily 1 h heat shocks for another 4 days. Progeny was then raised to adulthood, males collected and crossed to females containing marked balancer chromosomes. Single progeny males were then crossed individually to virgin females to generate additional offspring for PCR confirmation analysis and to balance the stocks in an isogenic background. A full deletion of CFAP299 (CG8138) was generated by CRISPR/Cas9‐mediated genome editing (Port *et al*, 2014). Two guideRNAs were cloned in the pCFD4 vector, one for the start and one for the end of the coding sequence. Co‐injection with a DsRed rescue plasmid (pHD‐DsRed‐attP plasmid; Gratz *et al*, 2015) with 1,000 bp flanking sequence on either side of the gene was carried out in vas‐Cas9 flies (BDSC #55821) by BestGene Inc. DsRed‐containing flies were screened by PCR to ensure proper targeting. The GFP fusion to Cep135/Bld10 under the control of the Ubiquitin promoter (Roque *et al*, 2012) was a gift of the Raff laboratory. Tex9 (CG4681) GFP was a FlyFos construct (Sarov *et al*, 2016) from the VDRC (#318643). All other GFP strains were generated as described previously (Stevens *et al*, 2010) using Ubq‐GFP plasmids for N and C‐terminal tagging. In brief, P element‐mediated transformation vectors containing GFP fusions to Cables1 (CG6191), CCDC6 (CG6664), Cep104 (CG10137), CFAP97D1 (CG14551), Elmod (CG10068), and Mapk15 (CG32703) were generated as follows: The complete coding region of each protein was amplified from cDNA with att sites at either end for Gateway cloning (Invitrogen). These fragments were inserted into the Gateway pDONR Zeo vector. The pDONR vectors were then recombined with Ubq plasmids (Peel *et al*, 2007), with each coding sequence placed in‐frame with GFP at the N or C terminus. The transgenic lines were generated by BestGene Inc. The Ubq promoter drives moderate expression in all tissues (Lee *et al*, 1988). Expression of N and C‐terminal constructs was examined in testes and neurons.

#### Method details

##### Phylogenetic profiling

Genomes were selected as widely as possible from all branches of the eukaryotic tree, taking care to avoid overrepresenting certain branches (See Dataset EV1A). In addition, a number of bacterial and archaeal genomes were included as outgroups. Only completely sequenced and well‐annotated genomes were chosen by confirming the presence of universally conserved genes. Starting point for our analysis was the human proteome, obtained from UNIPROT on 7 Feb 2014. Datasets were generated by performing a reciprocal BLASTp analysis for each human protein‐coding gene against the proteomes of each of the 159 genomes in our analysis. The BLAST algorithm used was NCBI BLAST 2.2.28, build 12 Mar 2013. If a hit was found above the EXPECT threshold of 0.1, the highest scoring protein sequence in this proteome was then run against the human proteome. If this reciprocal BLAST returned a protein of the same GeneID, a reciprocal hit was established for this protein, in that species, represented by a 1 in the output dataset. Otherwise, a 0 was reported for this protein‐species test. The resulting binary matrix was then used as input for hierarchical clustering in Gene Cluster, using the Euclidean distance metric and average linkage clustering for both genes and species on the entire dataset comprising 21,732 human protein‐coding genes and 159 species. Within the clustering output viewed in Java TreeView, a group of genes was identified with a similar phylogenetic inheritance pattern centered around the core centriolar component CENPJ/SAS‐4 (Kirkham *et al*, 2003). This cluster appeared quite distinct, such that loosening similarity constraints would only gradually grow the cluster until a final size of 386 genes was reached. This cluster was found to be highly enriched in known ciliary genes and hence was chosen for further analysis. To evaluate the robustness of our method, we repeated the hierarchical clustering with reduced numbers of species (25%, 50%, or 75% fewer), with genomes removed equally from all branches of the eukaryotic tree (see Dataset EV1A). The resultant clusters of genes centered around CENPJ were then compared with the original, full ciliary cluster and any differences in gene number and identity noted.

##### Identification of *C. elegans* and *Drosophila* orthologs

*C. elegans* and *Drosophila* orthologs of genes within the ciliary cluster were manually identified by reciprocal BLAST analysis using the human protein as the starting point. Where direct comparisons failed to identify a clear homolog, indirect searches were performed using less divergent related species as intermediates. To resolve gene duplications in one or other species, multiple sequence alignments were generated using MUSCLE within Jalview (http://www.jalview.org), with average distance phylogenetic trees constructed also in Jalview using the BLOSUM62 matrix.

##### Literature searches and database analyses

For each of the 386 genes in the cluster, we conducted a comprehensive literature and database analysis, including any information on their putative orthologs in nonvertebrate experimental models. For literature searches in PubMed, we employed all alternative names and classified each gene based on its level of functional characterization overall and any previously reported link to cilia. We further set out to provide a short description of its proposed function, with broader and more specific classifiers (e.g., centriole/cilium biogenesis and IFT‐A complex), including references to the relevant literature with PubMed identifiers. Any potential disease association relevant to ciliary function was reported separately, compiling reports from PubMed and OMIM (https://omim.org). Database analyses were conducted by retrieving the relevant datasets from the original source publication or website as indicated. To ensure proper comparisons, all identifiers were converted into Ensembl Gene IDs and their presence in the full human proteome dataset verified manually. The same was done with the sets of genes associated with specific centrosomal/ciliary functional modules, compiled from recent reviews and primary research publications. Classification of genes into predicted cilium biogenesis/motility genes was based on degree of conservation in ciliated/nonciliated species, presence in species with nonmotile cilia, species with a reduced complement of ciliary genes and *Plasmodium*, with cutoffs chosen manually based on the observed pattern for known ciliary genes (see Dataset EV2D).

##### *C. elegans* primary screen

Dye‐fill assays were performed on L4 stage animals. For each condition, ~ 100 worms were picked into 250 μl of M9 0.1% Triton, washed 3× with M9 0.1% Triton, 1× with M9, and then incubated at room temperature under dark conditions for 1 h in 0.2% DiI (Invitrogen) in M9. After incubation, worms were destained for at least 30 min on a seeded NGM plate before analysis. Dye accumulation in cell bodies of amphid and phasmid neurons was scored on a Zeiss Axio Imager Z2 microscope equipped with a 63× 1.4NA Plan‐Apochromat objective and Lumencor SOLA SE II light source. For illustration purposes, 0.2 μm z‐series were acquired on a DeltaVision 2 Ultra microscope, equipped with 7‐Color SSI module and sCMOS camera and controlled by Acquire Ultra acquisition software (GE Healthcare). Image stacks were deconvolved using Softworx and then imported into Fiji for postacquisition processing.

Dye‐fill phenotypes were assessed using the following scoring system: Amphids: 0 = 11.5–12 dye‐filling neurons, 1 = 11–11.49, 2 = 10–10.9, 3 = 6–9.9, 4 = 0–5.9, Phasmids: 0 = 3.85–4 dye‐filling phasmids, 1 = 3.75–3.84, 2 = 3.5–3.74, 3 = 0.25–3.49, 4 = 0–0.24. The strongest phenotype of amphids and phasmids was reported as the overall phenotype. Screening was carried out blind to the nature of the gene mutated in each assayed strain.

##### Further analysis of amphid and phasmid neurons in *C. elegans*

Live imaging of embryos and larvae was performed using a 60× 1.42NA Plan‐Apochromat objective on a DeltaVision 2 Ultra microscope, equipped with 7‐Color SSI module and sCMOS camera and controlled by Acquire Ultra acquisition software (GE Healthcare). Worms were anesthetized using 10 mM tetramisole in M9 for 5 min before being mounted on 2% agarose pads and imaged at room temperature. Embryos were mounted directly on agarose pads. 0.2 μm GFP/mCherry z‐series as well as single plane DIC images were collected for the amphid and phasmid neurons closest to the objective. For each condition and worm stage, at least 10 worms/embryos were examined. Image stacks were deconvolved using Softworx and then imported into Fiji for postacquisition processing. For strains expressing OSM‐6:GFP, 0.5 μm z‐series were acquired on a Zeiss Axio Imager Z2 microscope (described above) using a Photometrics CoolSNAP‐HQ2 cooled CCD camera controlled by ZEN 2 blue software (Zeiss).

Quantitation of phasmid dendrite lengths (from cell body to ciliary base) and ciliary length (from base to tip) was performed in Fiji using the Segmented Line tool. For each condition, a minimum of 20 worms were examined.

##### Transmission electron microscopy of *C. elegans*

L4‐stage worms were prepared using chemical fixation following the protocol described in Serwas & Dammermann (2015). Worms were fixed in 2.5% glutaraldehyde in cytoskeleton buffer (CB, 100 mM methyl ester sulfonate, 150 mM NaCl, 5 mM EGTA, 5 mM MgCl_2_, 5 mM glucose in ddH_2_O, pH 6.1) overnight at 4°C. Samples were then washed 3× in 1xCB and postfixed for 30 min in 0.5% osmium tetroxide in CB. Fixed worms were then washed 3× in CB and 1× in ddH_2_O. Finally, samples were dehydrated for 15 min each in 40, 60, 80%, 2× in 95%, and 3× in 100% acetone. Samples were embedded in Agar100 resin after fixation and dehydration. 70 nm serial sections were prepared onto Cu 100 mesh grids with Formvar film, then poststained with aqueous uranyl acetate and lead citrate, and examined with a Morgagni 268D microscope (FEI) equipped with an 11‐megapixel Morada CCD camera (Olympus‐SIS) and operated at 80 kV.

##### *Drosophila* primary screen

To deplete specific target genes in the whole fly or specific tissues, three different RNAi crosses were set up. We used Tub‐GAL4 as a ubiquitous driver. Tissue‐specific drivers used were as follows: a combination of Bam‐GAL4 (gift of Helen White‐Cooper, White‐Cooper, 2012) and Nanos‐GAL4 (Rørth, 1998) for germline depletion and Elav‐GAL4 (Lin & Goodman, 1994) for neuronal depletion. In each case, we crossed 5–8 virgins to four males from the respective RNAi strain (KK and GD: Dietzl *et al*, 2007, TRiP: Ni *et al*, 2008) in standard cornmeal‐agar vials. Vials were kept for 3 days at 25°C, 60% relative humidity, 12/12 light cycle. On the end of the third day, we discarded the parents and vials were moved to 29°C, 60% relative humidity, and 12/12 light cycle. Uncoordination, wing posture, and flight were scored in Elav and Tubulin crosses. Male fertility was scored in Bam/Nanos and Tubulin crosses. Viability was scored in all crosses. The strongest phenotype was reported from the different RNAi lines tested. Screening was carried out blind to the nature of the gene perturbed in each cross.

To test male fertility, we collected five males from the first cross and crossed them to 5 w‐ virgin females. As females do not lay eggs in the direct neighborhood of yeast granules, we used standard cornmeal vials without dry yeast to eliminate bias due to differences in yeast‐free surface availability. All fertility test crosses were kept at 29°C to strengthen knockdown efficiency. After 3 days, adults were removed and pupae scored 13 days after setting up the test cross with w‐ when all progeny had pupariated. Control crosses under these conditions produced on average 120 pupae. Pupa numbers were scored using the following scheme: 0 = 90 pupae or more, 1 = between 89 and 60, 2 = between 59 and 30, 3 = between 29 and 10, and 4 = less than 10.

Viability was scored comparing the number of dead larvae, dead pupae, and dead pigmented wing pharate adults to the overall progeny number. Viability was scored for all three stages. Few or no progeny was scored as larval lethality. To confirm early lethal phenotypes, crosses were repeated two to three times. The following scores were used: 4 = complete lethality, 3 = strong lethality with a few survivors, 2 = half of the progeny died at a given stage, 1 = dead individuals, but less than half of all offspring, 0 = no or few (< 3) dead individuals were observed. For sperm and neuron‐specific RNAi scores reported are a combination of pupal and pharate lethality.

Adult flies were analyzed using the following classification system to score uncoordination and flight. Uncoordination was scored as follows: 4 = Strong uncoordination with all adults sticking to the surface of the food, 3 = Uncoordinated flies at the bottom of the vial unable to climb up the vial. Weak uncoordination (2 and 1) was scored using a flight test. 20–30 flies were put into a fresh vial, and after 1 h of recovery, we tested the ability to fly away from the rim of the vial. Flight was scored as follows: 2 = more than half of the flies were unable to fly, 1 = less than half of the flies were unable to fly (> 3 no fliers). As wing defects affected flying ability, only flies without wing defects were used for flight test.

Wing posture defects scored were lowered wing blades, extended wings and completely erected or completely dropped wings. We scored the frequency of these phenotypes (> 20 counted): 4 = all individuals affected, 3 = more than half affected, 2 = half of flies affected, 1 = several individuals affected, 0 = No or few individuals affected (< 3).

##### *Drosophila* secondary screen—analysis of male germline defects

Fly strains were crossed as in the primary screen. 0–1 day‐old males were dissected in PBS. The tip of the abdomen with testis attached was placed in 4% formaldehyde in PBS for 25 min and washed 3 × 5 min in 0.1% PBS‐Triton‐X. Testes were transferred into a droplet of staining solution of 5 μg/ml Hoechst 33342 and a 1:50 dilution of Alexa 568 phalloidin in PBS for 2 h. Samples were washed overnight at 4°C in 1 ml of PBS. Testes of three independent males were mounted in mounting medium (2% w/v n‐propyl gallate in PBS/ 90% Glycerol) on multiwell slides, covered with a coverslip and imaged on a Zeiss LSM 700 confocal laser scanning microscope equipped with 25× Plan‐Apochromat oil immersion objective. Image stacks of approximately 20 z‐planes were acquired at 4 μm increments for each of the six testes. The maximum projections from 4 z‐stacks were stitched together using ZEN software (Zeiss) to cover the whole testis. Motility of sperm was tested by dissecting testes with seminal vesicle attached in a drop of Schneider media. The seminal vesicle was pierced with a sharp tungsten needle and sperm motility imaged immediately using dark field settings on a Zeiss Axio Imager Z2 microscope equipped with a 10× Plan‐Neofluar objective at 50 frames/s.

To classify defects, we distinguished six stages of spermiogenesis (Tates, 1971): Gonialblast stage (Tates stage 1, 2, 3), spermatocyte stage (large masses of heterochromatin at the periphery of the nucleus, Tates stage 4, 5, 6), spermatid stage with round nucleus (Tates stage 10–14), spermatid stage with elongated nucleus (Tates stage 17–18), and stacked/pinhead sperm/spermatid stage (Tates stage 19, 20). Additionally, we scored the number and shape of the actin cones during sperm individualization (marked by phalloidin‐positive membrane investment cones). Defects were scored for each stage using the following scale: 0 = normal morphology, 1 = weak deviation (occasionally observed in negative controls), 2 = stronger effect but normal morphology also observed, 3 = all or vast majority of nuclei/investment cones for a given stage show aberration, and 4 = missing stage. Motility was assessed visually based on the number of motile sperm and frequency of flagellar beat compared with wild‐type sperm. Absence of sperm from the seminal vesicle was also noted.

##### *Drosophila* secondary screen—analysis of defects in chordotonal neurons by DIC microscopy

Chordotonal organ samples were taken from P9 stage male puparia (Bainbridge & Bownes, 1981). Puparia were removed from the side of the vial with a pair of forceps and glued ventral side down to a slide covered with double‐sided adhesive tape. The operculum was removed and puparial case cut open along the dorsal midline. Pharate adults were taken out of the case and glued dorsal side down on the tape. Front legs were torn off and mounted on a slide in a droplet of PBS and covered with a cover slip. Gentle pressure was applied on the coverslip to expel the chordotonal organ from the femur. At least three chordotonal organs from three different individuals were imaged for each genotype on a Zeiss Axio Imager Z2 microscope equipped with 63X Plan‐Apochromat oil immersion objective in DIC modus. For each organ, a stack of 20 z‐planes was acquired at 0.5 μm increments.

The primary unit for quantification was the scolopidium, which contains two ciliated neurons. Scolopidia are organized in three groups (1, 2 and 3, Shanbhag *et al*, 1992). We only used scolopidia from groups 2 and 3. For morphological assessment and numerical quantification, three chordotonal organs from three different individuals were imaged and analyzed. Screening for dilation defects was done on all images for a given genotype. Defects in scolopidium morphology were observed and scored in the following way: “No cilium”—cilium not detected, “Short cilium”—tip does not reach the cap cell, “Regressive cilium”—thinner, bent ciliary shaft and “Vesicles in scolopidial space.” To measure the strength of each phenotype, the ratio of morphologically defective scolopidia compared with the total number of scolopidia of a given chordotonal organ was calculated. A gene was scored as abnormal if more than 20% of scolopidia contained neurons that were morphologically defective. To analyze the presence or absence of cilia, we counted the number of scolopidia with no cilium, 1 cilium, 2 cilia, or more than 2 cilia. In addition, we assessed morphological defects and mispositioning of the dilations.

##### *Drosophila* secondary screen—analysis of defects in chordotonal neurons using Sas‐4 and NompC staining

Leg chordotonal organs were dissected from 36‐h‐old male pupae. Fixation and staining were done as previously described for testes (Martinez‐Campos *et al*, 2004). Briefly, legs were dissected in PBS. Leg chordotonal organs were placed between slide and coverslip and pushed out of the cuticle by applying gentle pressure. Slides were then snap‐frozen in liquid nitrogen. After recovering slides and removing the coverslip, samples were incubated in ice‐cold methanol for 5 min, followed by ice‐cold acetone for 2 min. Samples were then washed twice with PBS 0.5% Triton X‐100, followed by blocking in PBS 0.1% Triton X‐100 1% BSA for 30 min. Primary antibodies for NompC and Sas‐4 were diluted 1:500 in blocking solution and added to the samples and incubated overnight at 4°C. The next day samples were washed twice with PBS before addition of secondary antibody in PBS for 2 h at room temperature. After washing once with PBS, Hoechst 33342 was added for 10 min. Samples were air‐dried and mounted with mounting medium. Defects for Sas‐4 staining (absence) and NompC staining (absence, misposition, and redistribution) were scored. Three different legs were analyzed and the average defect per scolopidium reported.

##### Detailed analysis by immunofluorescence microscopy in *Drosophila*

For detailed examination of sperm centrioles and cilia, testes were dissected from 72‐h‐old male pupae. Fixation and staining were done essentially as described above for chordotonal organs. After dissection, testes were transferred onto a microscope slide and cut with a tungsten needle before placing on a coverslip. Immunofluorescence samples were analyzed on a Zeiss LSM980 scanning confocal microscope equipped with an Airyscan unit. Stacks of 0.75 μm slices were acquired with a 63× 1.4NA Plan‐Apochromat lens using single‐channel mode to avoid cross‐illumination. Airyscan images were acquired for selected centrioles using 0.25 μm slices and 10× electronic magnification. Maximum intensity projections of Z‐stacks prepared in ImageJ were used for image analysis and panel preparation.

To quantitate localization interdependencies, the combined integrated signal intensity was measured for two regions encompassing the two basal bodies at the spermatocyte stage and the ratio of GFP signal to that of the countermarker (Ana1/γ‐tubulin) calculated for each pair. Plotted on the graphs is the average of the ratios for all basal body pairs normalized to the respective wild‐type control.

##### Transmission electron microscopy of *Drosophila*

To examine ultrastructure of chordotonal organs, legs from 36‐h‐old pupae were cut off with microscissors and fixed using a mixture of 2% glutaraldehyde and 2% paraformaldehyde in 0.1 mol/l sodium phosphate buffer, pH 7.2 for 2 h in a desiccator at room temperature and then overnight on a rotator at 4°C. Legs were then rinsed with sodium phosphate buffer, postfixed in 2% osmium tetroxide in buffer on ice, dehydrated in a graded series of acetone on ice, and embedded in Agar 100 resin. 70 nm sections were cut and poststained with 2% uranyl acetate and Reynolds lead citrate. Sections were examined with a Morgagni 268D microscope (FEI, Eindhoven, the Netherlands) operated at 80 kV. Images were acquired using an 11 megapixel Morada CCD camera (Olympus‐SIS).

To examine sperm flagellar ultrastructure, late pupal testes from third instar larvae were dissected in PBS and fixed using 2.5% glutaraldehyde in 0.1 mol/l sodium phosphate buffer, pH 7.2 for 1 h at room temperature. Samples were then rinsed with sodium phosphate buffer, postfixed in 2% osmium tetroxide in dH2O on ice, dehydrated in a graded series of acetone, and embedded in Agar 100 resin. 70 nm sections were then cut and processed as above.

#### Statistical analysis

All error bars are mean with standard deviation. Unpaired *t*‐tests were conducted using GraphPad Prism to compare different conditions in a specific experiment. For dendrite lengths (not normally distributed in certain mutant conditions), Wilcoxon Mann–Whitney tests were performed to compare experimental conditions. *, **, *** represent *P*‐values of <0.05, 0.01, and 0.001, respectively. Unless otherwise stated, tests are comparing indicated condition to control.

## Author contributions

**Jeroen Dobbelaere:** Conceptualization; formal analysis; investigation; visualization; writing – review and editing. **Tiffany Y Su:** Formal analysis; investigation; visualization; writing – review and editing. **Balazs Erdi:** Formal analysis; investigation; visualization. **Alexander Schleiffer:** Investigation. **Alexander Dammermann:** Conceptualization; formal analysis; writing – original draft; writing – review and editing.

## Disclosure and competing interests statement

The authors declare that they have no conflict of interest.

## Supporting information



Expanded View Figures PDF

Dataset EV1

Dataset EV2

Dataset EV3

Source Data for Expanded View

PDF+

Source Data for Figure 3

Source Data for Figure 4

Source Data for Figure 5

Source Data for Figure 6

Source Data for Figure 7

## Data Availability

The published article includes all datasets generated in this study. Source images for both main and Expanded View figures have been uploaded to the BioImage Archive (https://www.ebi.ac.uk/biostudies/bioimages/studies/S-BIAD681). *C. elegans* and *Drosophila* strains and plasmids generated in this study are available upon request from the corresponding author.
