# Supplementary material for: A phylogenetic profiling approach identifies novel ciliogenesis genes in Drosophila and C. elegans
Source: EMBO J. 2023 Jun 15;42(16):e113616. doi: 10.15252/embj.2023113616 (PMC10425847; doi:10.15252/embj.2023113616)
Supplement: Supplementary file 1 — Expanded View Figures PDF [file EMBJ-42-e113616-s007.pdf]

# Expanded View Figures

**Figure EV1. Further characterization of *C. elegans* MAPK-15 and ELMD-1.**

A Detailed examination of MAPK-15/ELMD-1 localization in *C. elegans* phasmid neurons. Endogenous promoter GFP:MAPK-15 co-localizes with the basal body marker HYLS-1 and accumulations of the IFT marker CHE-11, just proximal to the transition zone marked by MKS-6. A second population of MAPK-15 localizes to the adhesion belt at the proximal end of the periciliary membrane compartment. Endogenously GFP-tagged ELMD-1 localizes to the entire periciliary membrane compartment.

B Analysis of MAPK-15/ELMD-1 recruitment during *C. elegans* embryogenesis. MAPK-15 signal first becomes detectable in postmitotic sensory neurons marked by expression of a plasma membrane marker at the comma stage of embryogenesis (430 min after fertilization, Sulston *et al*, 1983), with ELMD-1 signal following shortly thereafter at the 1.5-fold stage (460 min), both proteins localizing to the distal tip of the elongating dendrite where the cilium will eventually form (Nechipurenko *et al*, 2017; Serwas *et al*, 2017). Neither GFP fusion is detectable at earlier stages of development or in other tissues of the worm. The hazy fluorescence signal in late stage embryos is due to autofluorescence. $n > 10$ animals for each developmental stage.

Data information: Scale bars are 10 µm (A, B), 5 µm (A, B, insets).

 © 2023 The Authors

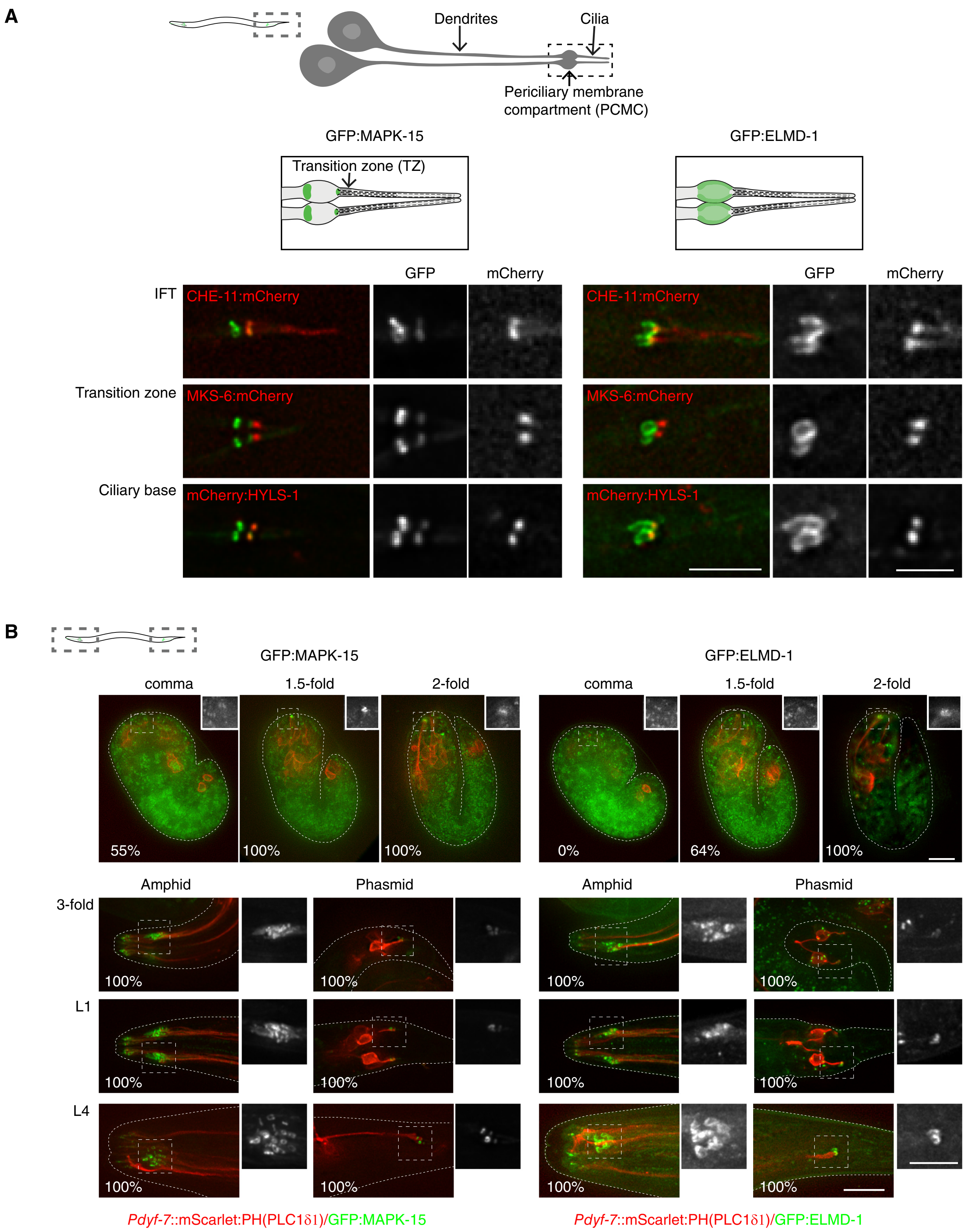
A
Dendrites
Cilia
Periciliary membrane compartment (PCMC)
GFP:MAPK-15
Transition zone (TZ)
GFP:ELMD-1
GFP
mCherry
IFT
CHE-11:mCherry
Transition zone
MKS-6:mCherry
Ciliary base
mCherry:HYLS-1
B
GFP:MAPK-15
GFP:ELMD-1
comma
1.5-fold
2-fold
55%
100%
0%
64%
Amphid
Phasmid
3-fold
L1
L4
*Pdyf-7*::mScarlet:PH(PLC1δ1)/GFP:MAPK-15
*Pdyf-7*::mScarlet:PH(PLC1δ1)/GFP:ELMD-1

**Figure EV1.**

**Figure EV2. Characterization of Mapk15 and Elmod in *Drosophila*.**

A, B RNAi-mediated depletion of MAPK15 and ELMOD in *Drosophila* sperm (A) and chordotonal neurons (B) results in defects in ciliary ultrastructure in both cellular contexts, with shortened (asterisks) and broken (arrowheads) axonemes in neurons and sperm, respectively. At least three animals examined per condition.

C GFP fusions to MAPK15 and ELMOD do not localize to centrioles marked by Ana1, but are recruited to maturing basal bodies during *Drosophila* spermatogenesis.

D MAPK15 and ELMOD co-localize with the centriolar marker Sas-4 on mature basal bodies in chordotonal neurons.

Data information: Scale bars are 100 μm (A, B), 1 μm (C, D). Error bars in (A) are standard deviation.
Source data are available online for this figure.

 © 2023 The Authors

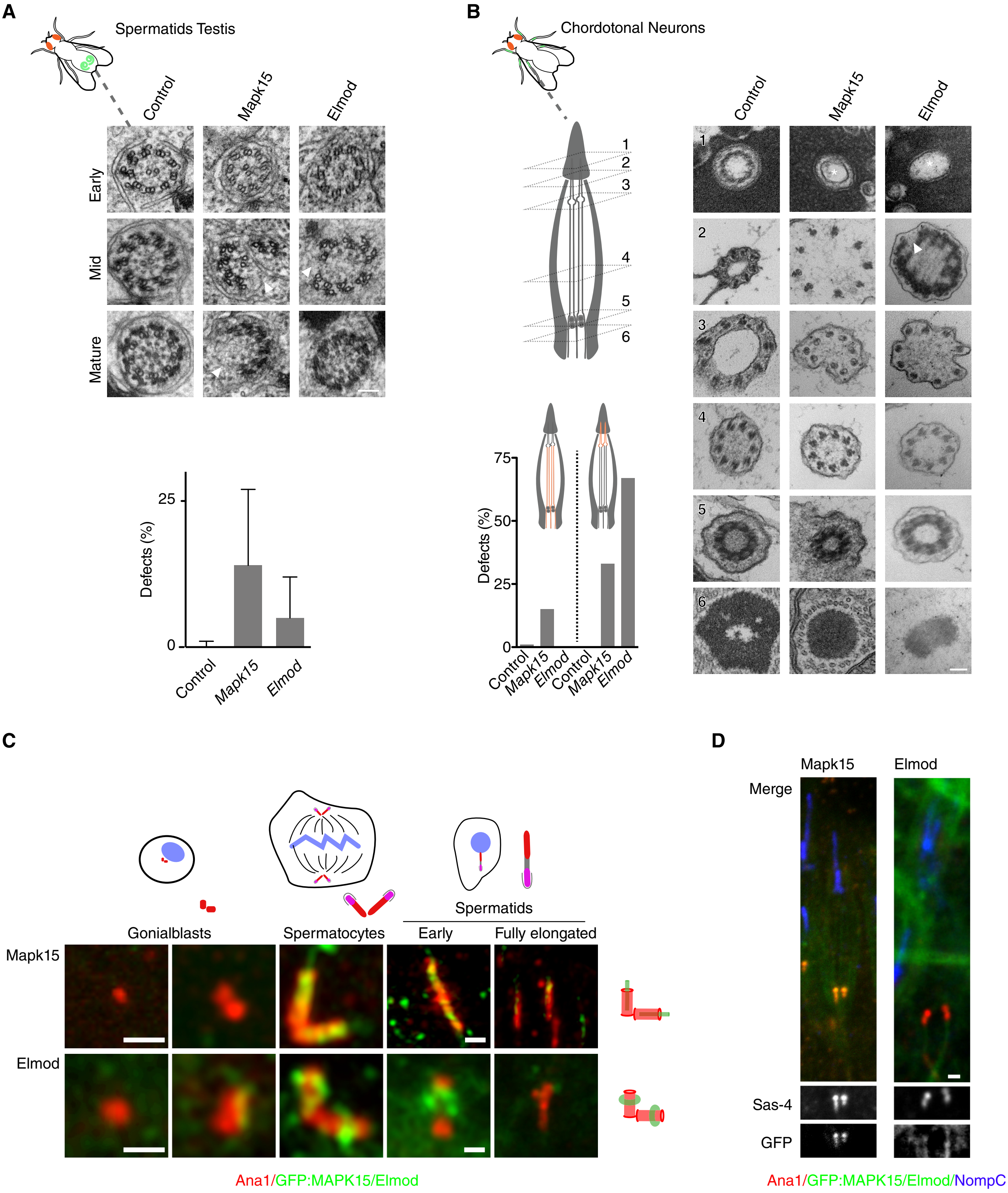
A
Spermatids Testis
Control
Mapk15
Elmod
Early
Mid
Mature
Defects (%)
25
0
Control
Mapk15
Elmod
B
Chordotonal Neurons
1
2
3
4
5
6
Control
Mapk15
Elmod
Defects (%)
75
50
25
0
Control
Mapk15
Elmod
Control
Mapk15
Elmod
C
Gonialblasts
Spermatocytes
Spermatids
Early
Fully elongated
Mapk15
Elmod
Ana1/GFP:MAPK15/Elmod
D
Mapk15
Elmod
Merge
Sas-4
GFP
Ana1/GFP:MAPK15/Elmod/NompC

**Figure EV2.**
