# Supplementary material for: A phylogenetic profiling approach identifies novel ciliogenesis genes in Drosophila and C. elegans
Source: EMBO J. 2023 Jun 15;42(16):e113616. doi: 10.15252/embj.2023113616 (PMC10425847; doi:10.15252/embj.2023113616)
Supplement: Supplementary file 12 [file EMBJ-42-e113616-s008.pdf]

THE EMBO JOURNAL

# A Phylogenetic Profiling Approach Identifies Novel Ciliogenesis Genes In Drosophila And C. elegans

Jeroen Dobbelaere, Tiffany Su, Balazs Erdi, Alexander Schleiffer, and Alexander Dammermann
**DOI: 10.15252/embj.2023113616**

*Corresponding author(s): Alexander Dammermann (alex.dammermann@univie.ac.at)*



## Review Timeline:

| | |
|---|---|
| Submission Date: | 26th Jan 23 |
| Editorial Decision: | 6th Mar 23 |
| Revision Received: | 27th Apr 23 |
| Editorial Decision: | 17th May 23 |
| Revision Received: | 22nd May 23 |
| Accepted: | 1st Jun 23 |

*Editor: Ieva Gailite*


## Transaction Report:

(Note: With the exception of the correction of typographical or spelling errors that could be a source of ambiguity, letters and reports are not edited. Depending on transfer agreements, referee reports obtained elsewhere may or may not be included in this compilation. Referee reports are anonymous unless the Referee chooses to sign their reports.)

**1st Editorial Decision** **6th Mar 2023**

Dear Dr. Dammermann,

Thank you for submitting your manuscript for consideration by the EMBO Journal. We have now received comments from three reviewers, which are included below for your information.

As you will see from the reports, the reviewers appreciate the work, while also indicating a number of constructive points for the improvement of the manuscript that would need to be addressed before acceptance here. From my side, I find the reviewer comments reasonable. Therefore, based on these positive assessments, I would like to invite you to address the issues raised by the reviewers in a revised manuscript. I would be happy to discuss the revision in more detail via email or phone/videoconferencing.

We generally allow three months as standard revision time. As a matter of policy, competing manuscripts published during this period will not negatively impact on our assessment of the conceptual advance presented by your study. However, please contact me as soon as possible upon publication of any related work to discuss the appropriate course of action. Should you foresee a problem in meeting this three-month deadline, please contact us to arrange an extension.

When preparing your letter of response to the referees' comments, please bear in mind that this will form part of the Review Process File and will therefore be available online to the community. For more details on our Transparent Editorial Process, please visit our website: https://www.embopress.org/page/journal/14602075/authorguide#transparentprocess. Please also see the attached instructions for further guidelines on preparation of the revised manuscript.

Please feel free to contact me if you have any further questions regarding the revision. Thank you for the opportunity to consider your work for publication, and I look forward to your revision.

With best regards,

Ieva

---
Ieva Gailite, PhD
Senior Scientific Editor
The EMBO Journal
Meyerhofstrasse 1
D-69117 Heidelberg
Tel: +4962218891309
i.gailite@embojournal.org

Instructions for preparing your revised manuscript:

Please make sure you upload a letter of response to the referees' comments together with the revised manuscript.

Please also check that the title and abstract of the manuscript are brief, yet explicit, even to non-specialists.

When assembling figures, please refer to our figure preparation guideline in order to ensure proper formatting and readability in print as well as on screen:
https://bit.ly/EMBOPressFigurePreparationGuideline
See also guidelines for figure legends: https://www.embopress.org/page/journal/14602075/authorguide#figureformat

At EMBO Press we ask authors to provide source data for the main manuscript figures. Our source data coordinator will contact you to discuss which figure panels we would need source data for and will also provide you with helpful tips on how to upload and organize the files.

IMPORTANT: When you send the revision we will require
- a point-by-point response to the referees' comments, with a detailed description of the changes made (as a word file).
- a word file of the manuscript text.
- individual production quality figure files (one file per figure)
- a complete author checklist, which you can download from our author guidelines (https://www.embopress.org/page/journal/14602075/authorguide).
- Expanded View files (replacing Supplementary Information)
Please see out instructions to authors

https://www.embopress.org/page/journal/14602075/authorguide#expandedview

Please remember: Digital image enhancement is acceptable practice, as long as it accurately represents the original data and conforms to community standards. If a figure has been subjected to significant electronic manipulation, this must be noted in the figure legend or in the 'Materials and Methods' section. The editors reserve the right to request original versions of figures and the original images that were used to assemble the figure.

Further information is available in our Guide For Authors: https://www.embopress.org/page/journal/14602075/authorguide

We realize that it is difficult to revise to a specific deadline. In the interest of protecting the conceptual advance provided by the work, we recommend a revision within 3 months (4th Jun 2023). Please discuss the revision progress ahead of this time with the editor if you require more time to complete the revisions.

------------------------------------------------

Referee #1:

In this work, Dobbelaere et al. performed comparative genomics on a diverse set of 147 eukaryotes, comparing those with and without cilia/flagella, to identify a phylogenetic inheritance pattern. The analysis revealed a cluster of 386 genes, centered on a core centriolar protein SAS4, that is highly enriched for known ciliary genes. Using C. elegans knockout and Drosophila depletion (RNAi) models, together with various assays of sensory and motile cilia structure and function, the authors found evidence of cilia-related roles for 50-88% of the novel genes within the ciliary cluster (37 assessed in worms; 68 assessed in flies). Finally, the authors selected several of the novel ciliary genes for more detailed functional analyses using TEM ultrastructure & protein localisation assays. Their data reveal CABLES1, CCDC6 and TEX9 as CEP135-related components that function at the basal body, CFAP97D1 as an axonemal assembly or stabilising component, and CFAP299 as a regulator of axonemal membrane deposition. The authors also identify a pathway involving MAPK15 and ELMOD that functions at the ciliary base region to regulate basal body/transition organisation and axonemal extension.

The work is very well executed and the data appears robust. The paper is also extremely well written and presented. Although comparative genomics has been used previously to identify new ciliary components (eg. two 2004 papers published in Cell), the present work considers a wider range of genomes and a more relaxed orthology inference to reduce the number of false negatives. Certainly, the 'ciliary gene cluster' looks comprehensive, containing many/most of the expected genes, and the authors have clearly demonstrated the ability to find novel ciliary genes within this dataset, and expand on their mechanisms of action. Although several papers have previously described ciliary base and PCMC-associated roles for MAPK15, the current work clearly extends the prior knowledge. Overall, I think this work will be of great interest to the cilia research community.

I only have a few minor suggestions:

1. The TEM images in Figures 5 and 6 could use some annotation to point out the specific defects in knockdown flies.

2. Some data lack quantification. For example, 5D-F.

3. Loss of elmd-1 causes increased levels of MAPK-15 at the BB and PCMC; does this not argue against the former being downstream of the latter, as the authors propose? Both genes are clearly interdependent in some way.

4.The paper describes various 'endogenous' GFP fusion reporters. This should be clarified. What is meant by 'endogenous' ? If it refers to expression levels, then the only reporter that best satisfies this criteria is one where GFP is inserted in frame at the genomic locus of the gene. Whilst mosSCI-derived reporters are expressed at levels that are close to endogenous, I am not sure you can say the reporter IS an endogenous reporter.

Referee #2:

This ambitious and clever study by Dobbelaer et al used phylogenic profiling of 147 diverse eukaryotes to identify core and new genes required for ciliogenesis and motility. While comparative genomic strategies have successfully identified ciliary genes, the strategy by Dammermann and colleagues identified 386 candidate primary/sensory and motile ciliary human genes, of which over 1/3rd are novel. To determine the function of these new genes, authors use the awesome power of genetic model systems Drosophila and C. elegans. Authors validate a cadre of new conserved ciliary genes with in vivo imaging/localization of fluorescently-tagged candidate and phenotypic analysis of mutant/tissue-specific RNAi-depleted animals using behavioral

analysis (fly), neuronal dye filling (worm), and in vivo imaging & TEM ultrastructural analysis (fly and worm). This dataset/paper is a gold-mine resource for those interested in development, ciliogenesis, and microtubules.

For those candidates with strong phenotypes and/or a compelling but uncharacterized evolutionary function in cilia, authors performed ultrastructural analysis and GFP-localization analysis. In Drosophila, authors show localization of 4-5 candidates to the spermatocyte basal body and show CEP135 is required for localization of one component (Ccdc6), but the newly identified components are not required for CEP135 localization (Figure 5F is difficult to interpret, see below). With C. elegans candidate validation, authors demonstrate a striking function for the MAPKKK MAPK-19 in both TZ and axonemal formation in worms then extend this dual role to ciliogenesis in chordontonal (compartmentalized) and spermatocytes (intracellular) cilia in flies. Another candidate, ELMOD, shares similar localization pattern and knockout/knockdown phenotypes with MAPK15 in both worms and flies. Double mutants look similar to single, with MAPK-19 being required for ELMD-1 localization but not vice versa. Genetic analysis is consistent with MAPK-19 acting upstream and providing a gratifying mechanism of action.

This manuscript will appeal to the broad readership of EMBO, with minor editorial revisions and suggested modifications to Figures to increase readability/understanding.
.
In order of the manuscript:

Abstract. "believe" - use another verb

General: Some parts of the manuscript were difficult to read, verbose, and complicated by use of parentheses (most of which were unnecessary). Authors are encouraged to edit and simplify sentence structure. For example:

In silico characterization of the ciliary phylogenetic cluster: this was a challenging section to read. Sentences were very long and used a lot of parentheses. This unnecessary complexity leads to problems understanding Figure 2E. I read the section and legend multiple times and am unclear on what the "unclear" category represents. Also, why can't a gene multi-task (ie biogenesis and motility functions)? Or a gene that functions in neither, for example, a signaling function? I think my Fig 2E confusion comes from the X-axis label, which should be non-motile vs motile cilia?

Figure 3C: Core biogenesis in Drosophila - this is not in the text and only in the legend. Core encompasses canonical and compartmentalized biogenesis? Again, the term biogenesis may too vague.

Figure 3F, G: Y-axis might be relabeled with a word for each value 0, 1, 2, 3, 4. For the 26 of 37 candidates that gave a weak dye fill defect, authors mention exhaustive Dyf screens for severe phenotypes. They might cite their Schouteden 2015 paper showing genetic modules/interactions between transition zone genes produce a more severe mutant phenotype in double and triple mutant combinations.

A simple supplemental table with a list of validated ciliary genes and phenotypes in Drosophila and/or C. elegans is warranted. One must wade through Supplemental Table 3 to extract this info.

In Figures 3 and 4, the cartoons of C. elegans and Drosophila anatomy are very helpful. The orientation of amphids and phasmids are different in left and right panels of Figure 3E, which might confuse a non-worm reader. Panels in Figure 4F aren't in the same order as the candidates examined in Figure 4G - it would be easier to interpret if 4G listed control, SAS4, CFAP299, MAPK15, Tex9, followed by the other candidates for which images are not shown.

Figure 5A-B, 6B, 6D, 7C, 7E please add labels to point out ultrastructural abnormalities for those readers not familiar with ciliary cross sections/TEM images. Links to higher resolution images are warranted.

Figure 5F: please add labels and possibly a cartoon to aid with interpretation.

Figure 7B: mapk-19, elmd-1, and the double mutant appear to have a bimodal distribution of dendritic extension defects: half look WT, half look severely Dex. Does this reflect differences in left-right patterning in the same animal?

Figure 7C: the control looks odd. WT should have 9 doublet microtubules in proximal ciliary regions - this panels show singlets and less than 9 in some cilia. Would authors please comment and/or show other representative images?

Fig 7D: what is the number of animals and number of amphid channel cilia examined?

The reviewer appreciates the accurate and comprehensive citations, including early foundational works.

Referee #3:

Cilia and flagella are highly organized structures that have been largely conserved during evolution. Despite two decades of extensive studies aimed at deciphering how these organelles are constructed, many building blocks are still missing to gain a complete understanding of some key steps in cilia formation. In this study, the authors revisit a genomic approach to identify conserved sets of ciliary components in ciliated species. Their approach is based on careful and accurate selection of 147 fully sequenced genomes that span all branches of the eukaryotic kingdom, taking care to avoid overrepresentation of certain branches, and scoring only the best reciprocal matches with the human proteome using a low-stringency Blastp threshold, followed by hierarchical clustering of genes on these scores. Strikingly, the authors identify a unique group of 386 genes including many known ciliary core components, but more importantly, they reveal 152 components as novel ciliary candidates, which, despite other published genomic studies with similar goals, is an important breakthrough.

To validate their list of candidates, the authors take advantage of two appropriate and complementary genetic models, flies and worms, to screen the entire list of candidates for ciliary function and demonstrate that more than 2/3 of the candidate genes are involved in ciliogenesis in either model. Last, genetic analysis in either organism highlights the functions of four novel protein groups: one is composed of Cables, Tek9, Ccdc6, Cep104 acting downstream of Cep135 in the centriolar lumen. All 5 contribute to the formation of the flagellar central pair. The second extends the motile function of the CFAP97 family of proteins as already revealed for some members of this family in human and fly. The third reveals a unique function of CFAP299 in ER/axoneme membrane organization in fly, and the last identifies a novel module composed of MAPK15 and ELMOD1 conserved in fly and worm that plays a critical role in both initiation of ciliogenesis and axoneme extension. Together, these results provide fascinating new functional ciliary modules that need to be integrated into the complex pathway of cilium assembly.

The data are robust and very well presented, and overall the conclusions are perfectly supported by the data.

I only have a few tiny suggestions for the ease of reading:

-Is it possible to add a table to summarize the number of genes that were actually tested in each model in the first and second screens and what the overlap is between candidates in flies (72 genes) and worms (56 genes). Could the authors also show in which subsets of the Venn diagram in Figure 3C are the genes that were studied in Figures 4-7 (although this data is available in the text or tables, it is not easy to follow). Related to this point are there really 226 dots on 3C and 136 on 3D, as the authors state all the genes were tested? And related to this point: could the authors indicate even more clearly how they selected the subset of genes for functional studies.

-Regarding CFAP299: the membrane deposition defect is apparently well ahead of the individualization process: this is ER membrane that ensheath the cytoplasmic axoneme. Surprisingly the actin cones are present and apparently normal (Figure 4F): is the image representative? Are the actin cones progressing?

-Elmod1 is downstream of Mapk15 in worms, is it also true in flies?

-EM sections of the chordotonal (5A, 6B, S2B): are the sections serial sections of the same neuron or representative images of different neurons? Page 15 end of the page "with missing axonemal doublets (Fig 5A): do you mean shorter cilia such as the doublets do not end as far as the control? One could understand odd number of doublets, which is not visible on the images.

-Figure 7E: please explain the arrowheads in the legend. Like for Drosophila: are the images serial sections of one neuron or representative images? It is not clear to me how you can see 60% of axoneme (indeed with odd symmetry) and 56% of completely disorganized ciliary base: is this because disorganization of the ciliary base is secondary/independent of axonemal growth?

-Figure 4B: arrows point to different details of the phenotype for each gene, please detail in the legend.

Page 22: "ELMOD and MAPK15 localisation was also very similar if not quite identical". This sentence is confusing. The localization is indeed at the ciliary base for the two genes in both organisms and ciliated tissues, but quite different between the two genes or between tissues. For example, in Drosophila the centriolar localization for Elmod and Mapk15 is very different in spermatocytes. It is also different in neurons. The end of the sentence should refer to S1A.

Page 18: "while staining for Hemingway" the sentence is correct but to avoid confusion add mature (as the protein is present along the axoneme until individualization but not in "mature" sperm).

Page 18: "a seemingly intact flagellar axoneme..." refers to Figure 6D (and not 6C).

**Response to Reviewers**

We would like to thank the editor and reviewers for their time and expert analysis of our work. We are very pleased that they see merit in our manuscript and the phylogenetic profiling-based screen it describes. Below please find our detailed point-by-point response to the reviewers' comments, along with those comments in full in italics. Any changes to the text or figures are highlighted in bold.

**Referee #1:**

*In this work, Dobbelaere et al. performed comparative genomics on a diverse set of 147 eukaryotes, comparing those with and without cilia/flagella, to identify a phylogenetic inheritance pattern. The analysis revealed a cluster of 386 genes, centered on a core centriolar protein SAS4, that is highly enriched for known ciliary genes. Using C. elegans knockout and Drosophila depletion (RNAi) models, together with various assays of sensory and motile cilia structure and function, the authors found evidence of cilia-related roles for 50-88% of the novel genes within the ciliary cluster (37 assessed in worms; 68 assessed in flies). Finally, the authors selected several of the novel ciliary genes for more detailed functional analyses using TEM ultrastructure & protein localisation assays. Their data reveal CABLES1, CCDC6 and TEX9 as CEP135-related components that function at the basal body, CFAP97D1 as an axonemal assembly or stabilising component, and CFAP299 as a regulator of axonemal membrane deposition. The authors also identify a pathway involving MAPK15 and ELMOD that functions at the ciliary base region to regulate basal body/transition organisation and axonemal extension.*

*The work is very well executed and the data appears robust. The paper is also extremely well written and presented. Although comparative genomics has been used previously to identify new ciliary components (eg. two 2004 papers published in Cell), the present work considers a wider range of genomes and a more relaxed orthology inference to reduce the number of false negatives. Certainly, the 'ciliary gene cluster' looks comprehensive, containing many/most of the expected genes, and the authors have clearly demonstrated the ability to find novel ciliary genes within this dataset, and expand on their mechanisms of action. Although several papers have previously described ciliary base and PCMC-associated roles for MAPK15, the current work clearly extends the prior knowledge. Overall, I think this work will be of great interest to the cilia research community.*

*I only have a few minor suggestions:*

*1. The TEM images in Figures 5 and 6 could use some annotation to point out the specific defects in knockdown flies.*

**Arrowheads now point at some of the key phenotypes seen in RNAi-depleted/mutant flies**. **We have further separated our quantitation of defects in the proximal/distal segment of the basal body/cilium in chordotonal neurons to better pinpoint the nature of defects observed (Figs. 5A, 6B, EV2B). Defects are further summarized in a new supplemental table (Table EV3C)**.

*2. Some data lack quantification. For example, 5D-F.*

**A quantitation of protein recruitment (Fig. 5D)/localization interdependencies (Fig. 5E, F) is now provided**. **In the case of MAPK-15/ELMD-1 localization interdependencies(Fig.**

**7G) a quantitation of signal intensities is difficult given the mislocalization of ELMD-1 in *mapk-15* mutants to other parts of the distal dendrite. We instead provide a quantitation of the number of phasmids displaying aberrant protein accumulation**.

*3. Loss of elmd-1 causes increased levels of MAPK-15 at the BB and PCMC; does this not argue against the former being downstream of the latter, as the authors propose? Both genes are clearly interdependent in some way.*

Negative feedback loops are not inconsistent with hierarchical recruitment pathways and indeed are not uncommon in this context. For example, centriolar Plk4 levels are kept in check by negative feedback loops involving downstream factors, with the ensuing oscillations helping to determine the site and timing of centriole assembly (Takao et al., Biol Open 2019; Aydogan et al., Cell 2020). The apparent role of ELMD-1 in limiting the accumulation of MAPK-15 at the ciliary base is therefore not incompatible with ELMD-1 functioning downstream of MAPK-15.

*4.The paper describes various 'endogenous' GFP fusion reporters. This should be clarified. What is meant by 'endogenous' ? If it refers to expression levels, then the only reporter that best satisfies this criteria is one where GFP is inserted in frame at the genomic locus of the gene. Whilst mosSCI-derived reporters are expressed at levels that are close to endogenous, I am not sure you can say the reporter IS an endogenous reporter.*

This manuscript utilizes both endogenously tagged (ie at the genomic locus of the gene) and endogenous promoter GFP reporters (ie using the 5' and 3' regulatory sequences of the gene to drive expression from a defined chromosomal insertion site). Which is which is specified in each case and our use of the latter terminology is standard in the field, distinguishing such transgenic reporters from those using heterologous tissue-specific promoters (Ppie-1, Posm-5, etc). In both cases the observed temporal and spatial expression pattern should accurately report on the behavior of the gene under study.

**Referee #2:**

*This ambitious and clever study by Dobbelaer et al used phylogenic profiling of 147 diverse eukaryotes to identify core and new genes required for ciliogenesis and motility. While comparative genomic strategies have successfully identified ciliary genes, the strategy by Dammermann and colleagues identified 386 candidate primary/sensory and motile ciliary human genes, of which over 1/3rd are novel. To determine the function of these new genes, authors use the awesome power of genetic model systems Drosophila and C. elegans. Authors validate a cadre of new conserved ciliary genes with in vivo imaging/localization of fluorescently-tagged candidate and phenotypic analysis of mutant/tissue-specific RNAi-depleted animals using behavioral analysis (fly), neuronal dye filling (worm), and in vivo imaging & TEM ultrastructural analysis (fly and worm). This dataset/paper is a gold-mine resource for those interested in development, ciliogenesis, and microtubules.*

*For those candidates with strong phenotypes and/or a compelling but uncharacterized evolutionary function in cilia, authors performed ultrastructural analysis and GFP-localization analysis. In Drosophila, authors show localization of 4-5 candidates to the spermatocyte basal body and show CEP135 is required for localization of one component (Ccdc6), but the newly identified components are not required for CEP135 localization (Figure 5F is difficult to interpret, see below). With C. elegans candidate validation, authors demonstrate a striking function for the MAPKKK MAPK-19 in both TZ and axonemal*

*formation in worms then extend this dual role to ciliogenesis in chordontonal (compartmentalized) and spermatocytes (intracellular) cilia in flies. Another candidate, ELMOD, shares similar localization pattern and knockout/knockdown phenotypes with MAPK15 in both worms and flies. Double mutants look similar to single, with MAPK-19 being required for ELMD-1 localization but not vice versa. Genetic analysis is consistent with MAPK-19 acting upstream and providing a gratifying mechanism of action.*

*This manuscript will appeal to the broad readership of EMBO, with minor editorial revisions and suggested modifications to Figures to increase readability/understanding.*

*In order of the manuscript:*

*Abstract. "believe" - use another verb*

**This has been changed to 'propose'**.

*General: Some parts of the manuscript were difficult to read, verbose, and complicated by use of parentheses (most of which were unnecessary). Authors are encouraged to edit and simplify sentence structure. For example:*

*In silico characterization of the ciliary phylogenetic cluster: this was a challenging section to read. Sentences were very long and used a lot of parentheses. This unnecessary complexity leads to problems understanding Figure 2E. I read the section and legend multiple times and am unclear on what the "unclear" category represents. Also, why can't a gene multi-task (ie biogenesis and motility functions)? Or a gene that functions in neither, for example, a signaling function? I think my Fig 2E confusion comes from the X-axis label, which should be non-motile vs motile cilia?*

We apologize for the confusion. In trying to make sense of the known genes within the ciliary cluster we find the broad classification into motility and biogenesis genes rather useful, with the former encompassing genes required exclusively for ciliary motility (inner and outer dynein arm components, dynein assembly factors, nexins, N-DRC, radial spoke and central apparatus components and microtubule inner proteins (MIPs)). Such genes would not be expected to be present in species with exclusively non-motile cilia. In contrast, any genes required for cilium biogenesis (including centriolar and transition zone components, IFT and BBS components) would be expected to be conserved in species with both motile and non-motile cilia. This is indeed what we found. The predictive power based on pattern of conservation declines with a more limited phylogenetic distribution of the ciliary cluster gene (with eg an absence of the gene in nematodes potentially simply reflecting poor conservation, not a function in motility), hence the category of 'unclear', where no confident prediction is possible.

In our classification, any function in assembly of immotile ciliary structures would place a 'multi-tasking' gene in the biogenesis category, regardless of any additional function in ciliary motility. This does seem appropriate. With motile and immotile cilia both having been found to have sensory functions, signaling genes would likewise fall into the biogenesis category. However, the ciliary cluster is largely devoid of signaling genes, reflecting the more recent evolutionary association of signaling pathways with cilia in the opisthokont lineage (Sigg et al., Dev Cell 2017). **We have added a sentence to the results section to draw attention to that fact and sought to better explain the *in silico* prediction in the relevant figure legend**.

*Figure 3C: Core biogenesis in Drosophila - this is not in the text and only in the legend. Core*

*encompasses canonical and compartmentalized biogenesis? Again, the term biogenesis may too vague.*

As with our classification of ciliary cluster genes into biogenesis and motility genes, we find the distinction between core and compartmentalized biogenesis genes to be a useful concept. The latter are required only in the context of canonical, IFT-dependent cilium assembly at the plasma membrane (including in *C. elegans* and *Drosophila* neurons), but are dispensable in the context of cytosolic cilium biogenesis (*Plasmodium*, *Drosophila* sperm), while core biogenesis genes function in both (there do not seem to be any genes specific to cytoplasmic assembly, suggesting this is the same basic process without the additional machinery required for assembly at the plasma membrane). This is the same classification first employed by Avidor-Reiss et al., Cell 2004, where the terms used were 'all ciliary' and 'compartment' genes. Interestingly, few genes appear to be truly universal (15 by our definition), including the centriolar structural components SAS-4/CENPJ and SAS6, but also MAPK15, underlining the importance of this regulator of ciliogenesis.

*Figure 3F, G: Y-axis might be relabeled with a word for each value 0, 1, 2, 3, 4. For the 26 of 37 candidates that gave a weak dye fill defect, authors mention exhaustive Dyf screens for severe phenotypes. They might cite their Schouteden 2015 paper showing genetic modules/ interactions between transition zone genes produce a more severe mutant phenotype in double and triple mutant combinations.*

We are not sure that a purely verbal description of phenotypic severity would be more accessible to the reader than a numerical scale, as long as each category is clearly defined. However, **in the interests of clarity we have added indicators of severity onto the graphs in Figs. 3B, C, F and G.** It is true that for the ciliary transition zone more severe phenotypes result from double/triple perturbations, as first noted by the Yoder lab (Williams et al., MBOC 2008, JCB 2011). However, to our knowledge no systematic enhancer screens have been performed to take advantage of this synergism. Consequently, genes scoring more weakly in the dyefill assay have been primarily identified based on homology to known ciliary genes in other species, although our work shows that even weak phenotypes are detectable using this assay given the invariance of dyefilling in wild-type *C. elegans*.

*A simple supplemental table with a list of validated ciliary genes and phenotypes in Drosophila and/or C. elegans is warranted. One must wade through Supplemental Table 3 to extract this info.*

**We have created a new supplemental table (Table EV3C) to collect together all information on the genes further characterized in our study**. This has also allowed us to provide a more detailed phenotypic description than is possible in the figures/text.

*In Figures 3 and 4, the cartoons of C. elegans and Drosophila anatomy are very helpful. The orientation of amphids and phasmids are different in left and right panels of Figure 3E, which might confuse a non-worm reader. Panels in Figure 4F aren't in the same order as the candidates examined in Figure 4G - it would be easier to interpret if 4G listed control, SAS4, CFAP299, MAPK15, Tex9, followed by the other candidates for which images are not shown.*

When depicting the head and tail of the worm we prefer to keep the same orientation as in the cartoon placed alongside each panel. **To clarify the position of cilia and cell bodies in the images of amphids and phasmids we have added explanatory labels where these are first shown (Fig. 3E)**.

As for the order of genes in Figure 4, in both panels B and C, as well as F and G, the idea was to separate controls and genes further studied in this manuscript, with the latter shown in

alphabetical order. In Fig. 4F we initially experimented with showing a more extensive set of controls, but then decided to limit ourselves to only SAS4 in order to be able to show some of the genes further characterized in our study without having to shrink images to indecipherable dimensions. However, the additional controls do serve a purpose in Fig. 4G to illustrate how different functional classes affect spermatogenesis at different stages.

*Figure 5A-B, 6B, 6D, 7C, 7E please add labels to point out ultrastructural abnormalities for those readers not familiar with ciliary cross sections/TEM images. Links to higher resolution images are warranted.*

**Arrowheads now highlight some of the key phenotypes seen in RNAi-depleted/mutant animals**. **Phenotypes are further summarized in Table EV3C**. As for image resolution, **source data are now provided for all images, including TEM images, as per EMBO Journal requirements**.

*Figure 5F: please add labels and possibly a cartoon to aid with interpretation.*

**The stages of spermatogenesis shown in the image panels are now indicated in the figure**.

*Figure 7B: mapk-19, elmd-1, and the double mutant appear to have a bimodal distribution of dendritic extension defects: half look WT, half look severely Dex. Does this reflect differences in left-right patterning in the same animal?*

If the reviewer is referring to a failure of dendrite extension preferentially in either the left or right set of phasmids, then no. There is no obvious left-right bias, nor is this generally seen in other mutants affecting the transition zone. The bimodal distribution of dendrite lengths itself (with not quite 50% more or less wild-type in length and the remainder fully collapsed) reflects the unusual nature of dendrite extension and the role of cilia in this process. As shown by the Shaham lab in 2009 (Heiman and Shaham, Cell), dendrite elongation in *C. elegans* amphids and phasmids occurs by retrograde extension, whereby it is the cell body that moves while the dendritic tip remains anchored in place via interactions with the surrounding extracellular matrix. As we subsequently showed in 2015 (Schouteden, Serwas et al., JCB), these interactions involve the ciliary transition zone. In transition zone mutants, failure of anchorage results in a stochastic failure of dendrite extension, an all or none phenotype occurring in a subset of neurons, albeit in a coordinate manner, with both phasmids in each pair losing anchorage simultaneously.

Given that dendrite lengths are clearly not normally distributed, a standard t-test to compare experimental conditions also appears inappropriate. **We therefore re-analysed these data using the non-parametric Wilcoxon Mann-Whitney test (Fig. 7B). This did not affect any of the results previously reported, in particular the significant differences observed between *mapk-15* and *elmd-1* single mutants and wild-type and the absence of any further enhancement in *mapk-15; elmd-1* double mutants compared to either single mutant.**

*Figure 7C: the control looks odd. WT should have 9 doublet microtubules in proximal ciliary regions - this panels show singlets and less than 9 in some cilia. Would authors please comment and/or show other representative images?*

This image is actually rather representative. Even in the most proximal region of the amphid channel singlet microtubules are seen in some cilia in wild-type animals - see Fig. 23D in the WormAtlas chapter on neuronal support cells:
https://www.wormatlas.org/hermaphrodite/neuronalsupport/mainframe.htm#NeuroFIG23

What can be assessed at this level is only the number of cilia extending into the amphid channel (invariably 10 in wild-type animals). Potential defects in axonemal architecture within cilia are consequently examined in the region below also containing the transition zones (E in Fig. 23D).

*Fig 7D: what is the number of animals and number of amphid channel cilia examined?*

**The full statistics for the number of animals/cilia examined in each figure are now provided as part of Table EV3C.**

*The reviewer appreciates the accurate and comprehensive citations, including early foundational works.*

Thank you. We have tried to be as comprehensive as possible in our citations, although given the large body of work on cilia and ciliogenesis this wasn't always easy. We also appreciate that The EMBO Journal does not enforce strict character/word limits on the main manuscript text which often hinder proper citation.

**Referee #3:**

*Cilia and flagella are highly organized structures that have been largely conserved during evolution. Despite two decades of extensive studies aimed at deciphering how these organelles are constructed, many building blocks are still missing to gain a complete understanding of some key steps in cilia formation. In this study, the authors revisit a genomic approach to identify conserved sets of ciliary components in ciliated species. Their approach is based on careful and accurate selection of 147 fully sequenced genomes that span all branches of the eukaryotic kingdom, taking care to avoid overrepresentation of certain branches, and scoring only the best reciprocal matches with the human proteome using a low-stringency Blastp threshold, followed by hierarchical clustering of genes on these scores. Strikingly, the authors identify a unique group of 386 genes including many known ciliary core components, but more importantly, they reveal 152 components as novel ciliary candidates, which, despite other published genomic studies with similar goals, is an important breakthrough.*

*To validate their list of candidates, the authors take advantage of two appropriate and complementary genetic models, flies and worms, to screen the entire list of candidates for ciliary function and demonstrate that more than 2/3 of the candidate genes are involved in ciliogenesis in either model. Last, genetic analysis in either organism highlights the functions of four novel protein groups: one is composed of Cables, Tek9, Ccdc6, Cep104 acting downstream of Cep135 in the centriolar lumen. All 5 contribute to the formation of the flagellar central pair. The second extends the motile function of the CFAP97 family of proteins as already revealed for some members of this family in human and fly. The third reveals a unique function of CFAP299 in ER/axoneme membrane organization in fly, and the last identifies a novel module composed of MAPK15 and ELMOD1 conserved in fly and worm that plays a critical role in both initiation of ciliogenesis and axoneme extension. Together, these results provide fascinating new functional ciliary modules that need to be integrated into the complex pathway of cilium assembly.*

*The data are robust and very well presented, and overall the conclusions are perfectly supported by the data.*

*I only have a few tiny suggestions for the ease of reading:*

*-Is it possible to add a table to summarize the number of genes that were actually tested in each model in the first and second screens and what the overlap is between candidates in flies (72 genes) and worms (56 genes). Could the authors also show in which subsets of the Venn diagram in Figure 3C are the genes that were studied in Figures 4-7 (although this data is available in the text or tables, it is not easy to follow). Related to this point are there really 226 dots on 3C and 136 on 3D, as the authors state all the genes were tested? And related to this point: could the authors indicate even more clearly how they selected the subset of genes for functional studies.*

**As also requested by reviewers 1 and 2, we have created a new supplemental table (Table EV3C) to collect together all information on the genes further characterized in our study**. **We have also marked those genes in Figs. 3C (*Drosophila*) and 3G (*C. elegans*)**. We did not quite test all 226 and 136 ciliary cluster genes conserved in *Drosophila* and *C. elegans*, respectively. Actual numbers were limited to the 218 genes in *Drosophila* with available RNAi lines and 105 genes in *C. elegans* for which loss of function mutants were available or were generated during the course of this study. Results for depletion/mutation of all of these genes are plotted in Figs. 3C and 3G. As for our rationale for selecting individual genes for further study, our primary screen suggested that essentially all genes within the cluster are likely cilium-related. We therefore chose genes which displayed particulary prominent phenotypes (**see also reviewer figure Fig. R1**) or where their pattern of conservation was consistent with a central role in ciliogenesis across eukaryotes. However, other criteria would have been equally valid and likely would have revealed other interesting genes for further study.

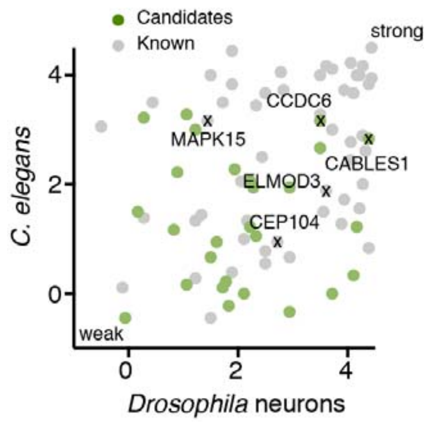


**Figure R1.** Comparison of primary screen phenotypes in *Drosophila* neurons and *C. elegans*, indicating conserved genes chosen for further study. Compare manuscript Figs. 3C and G.

*-Regarding CFAP299: the membrane deposition defect is apparently well ahead of the individualization process: this is ER membrane that ensheath the cytoplasmic axoneme. Surprisingly the actin cones are present and apparently normal (Figure 4F): is the image representative? Are the actin cones progressing?*

We would like to thank the reviewer for pointing out this apparent inconsistency. Actin cones require the membrane to form so should be absent if the membrane is not forming correctly. This was indeed the case for CFAP299: Out of the 8 testes examined, actin cones were entirely absent in 2 testes and severely reduced/malformed in the remaining 6. Actin cones were also defective in the case of MAPK15, in line with the quantitation presented in the figure. The

apparently normal phalloidin images for these two RNAi conditions were the result of incorrect panel assembly. **These image panels have now been corrected**.

*-Elmod1 is downstream of Mapk15 in worms, is it also true in flies?*

We have been unable to assess localization interdependencies in flies due to reagent limitations. Attempts to generate deletion mutants in ELMOD and MAPK15 in *Drosophila* have so far failed. While localization interdependency could in principle also be assessed by RNAi, this is more cumbersome. Moreover, ELMOD GFP signal at basal bodies/the ciliary base in *Drosophila* is not very robust. A further reduction in signal following MAPK15 depletion/ mutation would therefore not necessarily be obvious/conclusive.

*-EM sections of the chordotonal (5A, 6B, S2B): are the sections serial sections of the same neuron or representative images of different neurons? Page 15 end of the page "with missing axonemal doublets (Fig 5A): do you mean shorter cilia such as the doublets do not end as far as the control? One could understand odd number of doublets, which is not visible on the images.*

We have not performed serial sections in flies. What we meant by missing axonemal microtubule doublets in Fig. 5A is that these do not extend towards the ciliary tip (section 1 in the panel). **This is now clarified in the text.**

*-Figure 7E: please explain the arrowheads in the legend. Like for Drosophila: are the images serial sections of one neuron or representative images? It is not clear to me how you can see 60% of axoneme (indeed with odd symmetry) and 56% of completely disorganized ciliary base: is this because disorganization of the ciliary base is secondary/independent of axonemal growth?*

**The arrowheads in Fig. 7E are now better explained in the text**. Although we did perform serial sections in *C. elegans*, as in *Drosophila* the images shown for axoneme, transition zone and ciliary base are not necessarily from the same cilium but representative images to illustrate the main phenotypes. Regarding the progression of those phenotypes from base to tip, the main defect at the level of the degenerated basal body is membrane blebs, a phenotype we previously noted for loss of the acentriolar MTOC at the ciliary base, associated as here with axoneme elongation defects (Garbrecht, Laos et al., Curr Biol 2021). In the case of MTOC perturbation this was, however, not associated with fragmented central cylinders at the level of the transition zone, the main defect seen at this level for *mapk-15* and *elmd-1* mutants. Conversely, disorganization or complete loss of transition zone structures (Y-links, central cylinder) does not significantly affect axoneme elongation, as we showed in 2015 (Schouteden, Serwas et al., JCB). Given that transition zones in *mapk-15* and *elmd-1* mutants largely retain 9 doublet microtubules, the observed lack of 9-fold symmetry in the axoneme proper of *mapk-15* and *elmd-1* mutants points to at least two independent roles of MAPK-15/ELMD-1, in transition zone organization (1) and axoneme elongation (2).

*-Figure 4B: arrows point to different details of the phenotype for each gene, please detail in the legend.*

**We have now added further information in the figure legend.**

*Page 22: "ELMOD and MAPK15 localisation was also very similar if not quite identical". This sentence is confusing. The localization is indeed at the ciliary base for the two genes in both organisms and ciliated tissues, but quite different between the two genes or between tissues. For example, in Drosophila the centriolar localization for Elmod and Mapk15 is very different in spermatocytes. It is also different in neurons. The end of the sentence should refer to S1A.*

**The figure reference has been corrected**. Regarding ELMOD and MAPK15 expression and localization, to us the similarities outweigh the differences. Both proteins localize to basal bodies in *Drosophila* and the ciliary base and periciliary membrane compartment in *C. elegans* (the latter dual localization pattern not a common phenomenon in worms) and both proteins are recruited to basal bodies early in ciliogenesis with near-identical timing. It is clear that the proteins do not perfectly co-localize. However, the observed overlap is consistent with the proposed function of ELMOD downstream of MAPK15.

*Page 18: "while staining for Hemingway" the sentence is correct but to avoid confusion add mature (as the protein is present along the axoneme until individualization but not in "mature" sperm).*
**Done**.

*Page 18: "a seemingly intact flagellar axoneme..." refers to Figure 6D (and not 6C).*
**Figure reference has been corrected**.

Dear Dr. Dammermann,

Thank you for submitting a revised version of your manuscript. Your study has now been seen by one of the original referees, who finds that their previous concerns have been addressed and now recommends publication of the manuscript. There remain only a couple of editorial points that have to be addressed before I can extend formal acceptance of the manuscript:
1. Our publisher has done their pre-publication check on your manuscript. I have attached the file here. Please take a look at the word file and the comments regarding the figure legends and respond to the issues. Please also use this version when you resubmit the revised version.
2. Please rename the "Conflict of Interest" section into "Disclosure and competing interests statement"
3. Please rename the "Data and code availability" section into "Data availability".
4. Please move References after Figure and EV Figure legends.
5. Please rename Table EV1-EV3 into Dataset EV1-EV3 and correct the callouts and the corresponding legends.
6. Figure panels for Figure 5A and EV2B (control 2 panel) and Figure 7F and EV1 (CHE-11:Cherry) appear to be derived from the same sample. Please add a clarification in the figure legends.
7. CRediT has replaced the traditional author contributions section because it offers a systematic, machine-readable author contributions format that allows for more effective research assessment. Please remove the Authors Contributions from the manuscript and use the free text boxes beneath each contributing author's name in our online submission system to add specific details on the author's contribution. More information is available in our guide to authors.
8. Papers published in The EMBO Journal are accompanied online by a 'Synopsis' to enhance discoverability of the manuscript. It consists of A) a short (1-2 sentences) summary of the findings and their significance, B) 3-4 bullet points highlighting key results and C) a synopsis image that is 550x300-600 pixels large (width x height, jpeg or png format). You can either show a model or key data in the synopsis image. Please note that the image size is rather small and that text needs to be readable at the final size. Please send us this information together with the revised manuscript.

Please let me know if you have any questions regarding any of these points. You can use the link below to upload the revised files.

Thank you again for giving us the chance to consider your manuscript for The EMBO Journal. I look forward to receiving the final version.

With best regards,

Ieva

----
Ieva Gailite, PhD
Senior Scientific Editor
The EMBO Journal
Meyerhofstrasse 1
D-69117 Heidelberg
Tel: +4962218891309
i.gailite@embojournal.org

Further information is available in our Guide For Authors: https://www.embopress.org/page/journal/14602075/authorguide

We realize that it is difficult to revise to a specific deadline. In the interest of protecting the conceptual advance provided by the work, we recommend a revision within 3 months (15th Aug 2023). Please discuss the revision progress ahead of this time with the editor if you require more time to complete the revisions.

------------------------------------------------

Referee #3:

The authors have clarified all of the requested points, which makes it easier to understand the data. The EV3 table will certainly be helpful.

I no longer have any restrictions for publication.

The authors have addressed all minor editorial requests.

Dear Dr. Dammermann,

I apologise for the delay in handling of your manuscript due to my absence from the office last week and the resulting backlog. Thank you for addressing most of the final editorial issues. I am now pleased to inform you that your manuscript has been accepted for publication.

Before we forward your manuscript to our publishers, I would like to propose a couple of minor changes in the article abstract and synopsis. I have also written a short blurb that will accompany the title of your manuscript in our online table of contents. Please take a look at the text below and in the attached manuscript text file and let me know if any corrections are necessary.

Blurb:
Functional analysis of novel ciliary genes identifies CABLES1, CCDC6 and TEX9 as the components of a basal body module recruited by the cartwheel component Bld10/CEP135.

Synopsis:
Cilia are evolutionarily highly ancient structures, thought to date back to the last common ancestor of all eukaryotes. Here, a combination of comparative genomics and functional analysis in Drosophila and C. elegans was used to define and characterize the core set of genes involved in cilium assembly and motility across eukaryotes.

• Phylogenetic profiling defines a core set of 386 genes associated with cilium assembly and motility.
• Comprehensive tissue-specific depletion and mutant analysis confirms ciliary association of novel genes in Drosophila and C. elegans and reveals candidates for further study.
• CABLES1, CCDC6 and TEX9 are identified as novel components of a basal body module that is recruited by the centriolar cartwheel component Bld10/CEP135.
• MAPK15 and ELMOD are highly conserved regulators of multiple steps in the initiation of ciliogenesis.

Please note that it is EMBO Journal policy for the transcript of the editorial process (containing referee reports and your response letter) to be published as an online supplement to each paper. If you do NOT want this, you will need to inform the Editorial Office via email immediately. More information is available here:
https://www.embopress.org/page/journal/14602075/authorguide#transparentprocess

Your manuscript will be processed for publication in the journal by EMBO Press. Manuscripts in the PDF and electronic editions of The EMBO Journal will be copy edited, and you will be provided with page proofs prior to publication. Please note that supplementary information is not included in the proofs.

You will be contacted by Wiley Author Services to complete licensing and payment information. The required 'Page Charges Authorization Form' is available here: https://www.embopress.org/pb-assets/embo-site/tej_apc.pdf - please download and complete the form and return to embopressproduction@wiley.com

EMBO Press participates in many Publish and Read agreements that allow authors to publish Open Access with reduced/no publication charges. Check your eligibility: https://authorservices.wiley.com/author-resources/Journal-Authors/open-access/affiliation-policies-payments/index.html

Should you be planning a Press Release on your article, please get in contact with embojournal@wiley.com as early as possible, in order to coordinate publication and release dates.

If you have any questions, please do not hesitate to call or email the Editorial Office. Thank you again for this contribution to The EMBO Journal and congratulations on a successful publication!

Best regards,

Ieva

---
Ieva Gailite, PhD
Senior Scientific Editor
The EMBO Journal
Meyerhofstrasse 1
D-69117 Heidelberg
Tel: +4962218891309
i.gailite@embojournal.org

## EMBO Press Author Checklist

| | |
|---|---|
| Corresponding Author Name: Alexander Dammermann | |
| Journal Submitted to: The EMBO Journal | |
| Manuscript Number: EMBOJ-2023-113616 | |

**USEFUL LINKS FOR COMPLETING THIS FORM**
The EMBO Journal - Author Guidelines
EMBO Reports - Author Guidelines
Molecular Systems Biology - Author Guidelines
EMBO Molecular Medicine - Author Guidelines

**Reporting Checklist for Life Science Articles (updated January**
This checklist is adapted from Materials Design Analysis Reporting (MDAR) Checklist for Authors. MDAR establishes a minimum set of requirements in transparent reporting in the life sciences (see Statement of Task: 10.31222/osf.io/9sm4x). Please follow the journal's guidelines in preparing your
**Please note that a copy of this checklist will be published alongside your article.**

**Abridged guidelines for figures**
**1. Data**
The data shown in figures should satisfy the following conditions:
- the data were obtained and processed according to the field's best practice and are presented to reflect the results of the experiments in an accurate and unbiased manner.
- ideally, figure panels should include only measurements that are directly comparable to each other and obtained with the same assay.
- plots include clearly labeled error bars for independent experiments and sample sizes. Unless justified, error bars should not be shown for technical
- if n<5, the individual data points from each experiment should be plotted. Any statistical test employed should be justified.
- Source Data should be included to report the data underlying figures according to the guidelines set out in the authorship guidelines on Data

**2. Captions**
Each figure caption should contain the following information, for each panel where they are relevant:
- a specification of the experimental system investigated (eg cell line, species name).
- the assay(s) and method(s) used to carry out the reported observations and measurements.
- an explicit mention of the biological and chemical entity(ies) that are being measured.
- an explicit mention of the biological and chemical entity(ies) that are altered/varied/perturbed in a controlled manner.
- the exact sample size (n) for each experimental group/condition, given as a number, not a range;
- a description of the sample collection allowing the reader to understand whether the samples represent technical or biological replicates (including how many animals, litters, cultures, etc.).
- a statement of how many times the experiment shown was independently replicated in the laboratory.
- definitions of statistical methods and measures:
  - common tests, such as t-test (please specify whether paired vs. unpaired), simple χ2 tests, Wilcoxon and Mann-Whitney tests, can be unambiguously identified by name only, but more complex techniques should be described in the methods section;
  - are tests one-sided or two-sided?
  - are there adjustments for multiple comparisons?
  - exact statistical test results, e.g., P values = x but not P values < x;
  - definition of 'center values' as median or average;
  - definition of error bars as s.d. or s.e.m.

**Please complete ALL of the questions below.**
**Select "Not Applicable" only when the requested information is not relevant for your study.**

**Materials**

| Newly Created Materials | Information included in the manuscript? | In which section is the information available? (Reagents and Tools Table, Materials and Methods, Figures, Data Availability Section) |
|---|---|---|
| New materials and reagents need to be available; do any restrictions apply? | Yes | Materials and Methods |

| Antibodies | Information included in the manuscript? | In which section is the information available? (Reagents and Tools Table, Materials and Methods, Figures, Data Availability Section) |
|---|---|---|
| For **antibodies** provide the following information:<br>- Commercial antibodies: RRID (if possible) or supplier name, catalogue number and or/clone number<br>- Non-commercial: RRID or citation | Yes | Reagents and Tools Table |

| DNA and RNA sequences | Information included in the manuscript? | In which section is the information available? (Reagents and Tools Table, Materials and Methods, Figures, Data Availability Section) |
|---|---|---|
| **Short novel DNA or RNA including primers, probes**: provide the sequences. | Yes | Reagents and Tools Table |

| Cell materials | Information included in the manuscript? | In which section is the information available? (Reagents and Tools Table, Materials and Methods, Figures, Data Availability Section) |
|---|---|---|
| **Cell lines:** Provide species information, strain. Provide accession number in repository **OR** supplier name, catalog number, clone number, and/**OR** RRID. | Not Applicable | |
| **Primary cultures:** Provide species, strain, sex of origin, genetic modification status. | Not Applicable | |
| Report if the cell lines were recently **authenticated** (e.g., by STR profiling) and tested for mycoplasma contamination. | Not Applicable | |

| Experimental animals | Information included in the manuscript? | In which section is the information available? (Reagents and Tools Table, Materials and Methods, Figures, Data Availability Section) |
|---|---|---|
| **Laboratory animals or Model organisms:** Provide species, strain, sex, age, genetic modification status. Provide accession number in repository **OR** supplier name, catalog number, clone number, **OR** RRID. | Yes | Reagents and Tools Table |
| **Animal observed in or captured from the field:** Provide species, sex, and age where possible. | Not Applicable | |
| Please detail **housing and husbandry conditions**. | Yes | Materials and Methods |

| Plants and microbes | Information included in the manuscript? | In which section is the information available? (Reagents and Tools Table, Materials and Methods, Figures, Data Availability Section) |
|---|---|---|
| **Plants:** provide species and strain, ecotype and cultivar where relevant, unique accession number if available, and source (including location for collected wild specimens). | Not Applicable | |
| **Microbes:** provide species and strain, unique accession number if available, and source. | Not Applicable | |

| Human research participants | Information included in the manuscript? | In which section is the information available? (Reagents and Tools Table, Materials and Methods, Figures, Data Availability Section) |
|---|---|---|
| If collected and within the bounds of privacy constraints report on age, sex and gender or ethnicity for all study participants. | Not Applicable | |

| Core facilities | Information included in the manuscript? | In which section is the information available? (Reagents and Tools Table, Materials and Methods, Figures, Data Availability Section) |
|---|---|---|
| If your work benefited from core facilities, was their service mentioned in the acknowledgments section? | Yes | Acknowledgements |

**Design**

| Study protocol | Information included in the manuscript? | In which section is the information available? (Reagents and Tools Table, Materials and Methods, Figures, Data Availability Section) |
|---|---|---|

| | | |
|---|---|---|
| If study protocol has been **pre-registered, provide DOI in the manuscript**. For clinical trials, provide the trial registration number **OR** cite DOI. | Not Applicable | |
| Report the **clinical trial registration number** (at ClinicalTrials.gov or equivalent), where applicable. | Not Applicable | |

| Laboratory protocol | Information included in the manuscript? | In which section is the information available? (Reagents and Tools Table, Materials and Methods, Figures, Data Availability Section) |
|---|---|---|
| Provide DOI OR other citation details if **external detailed step-by-step protocols** are available. | Not Applicable | |

| Experimental study design and statistics | Information included in the manuscript? | In which section is the information available? (Reagents and Tools Table, Materials and Methods, Figures, Data Availability Section) |
|---|---|---|
| Include a statement about **sample size** estimate even if no statistical methods were used. | Yes | Materials and Methods |
| Were any steps taken to minimize the effects of subjective bias when allocating animals/samples to treatment (e.g. **randomization procedure**)? If yes, have they been described? | Not Applicable | |
| Include a statement about **blinding** even if no blinding was done. | Yes | Materials and Methods |
| Describe **inclusion/exclusion criteria** if samples or animals were excluded from the analysis. Were the criteria pre-established?<br><br>If sample or data points were omitted from analysis, report if this was due to attrition or intentional exclusion and provide justification. | Not Applicable | |
| For every figure, are **statistical tests** justified as appropriate? Do the data meet the assumptions of the tests (e.g., normal distribution)? Describe any methods used to assess it. Is there an estimate of variation within each group of data? Is the variance similar between the groups that are being statistically compared? | Yes | Materials and Methods |

| Sample definition and in-laboratory replication | Information included in the manuscript? | In which section is the information available? (Reagents and Tools Table, Materials and Methods, Figures, Data Availability Section) |
|---|---|---|
| In the figure legends: state number of times the experiment was **replicated** in laboratory. | Yes | Materials and Methods |
| In the figure legends: define whether data describe **technical or biological replicates**. | Not Applicable | |

## Ethics

| Ethics | Information included in the manuscript? | In which section is the information available? (Reagents and Tools Table, Materials and Methods, Figures, Data Availability Section) |
|---|---|---|
| Studies involving **human participants**: State details of **authority granting ethics approval** (IRB or equivalent committee(s), provide reference number for approval. | Not Applicable | |
| Studies involving **human participants**: Include a statement confirming that **informed consent** was obtained from all subjects and that the experiments conformed to the principles set out in the WMA Declaration of Helsinki and the Department of Health and Human Services Belmont Report. | Not Applicable | |
| Studies involving **human participants:** For publication of **patient photos**, include a statement confirming that consent to publish was obtained. | Not Applicable | |
| Studies involving experimental **animals**: State details of **authority granting ethics approval** (IRB or equivalent committee(s), provide reference number for approval. Include a statement of compliance with ethical regulations. | Not Applicable | |
| Studies involving **specimen and field samples:** State if relevant **permits** obtained, provide details of authority approving study; if none were required, explain why. | Not Applicable | |

| Dual Use Research of Concern (DURC) | Information included in the manuscript? | In which section is the information available? (Reagents and Tools Table, Materials and Methods, Figures, Data Availability Section) |
|---|---|---|
| Could your study fall under dual use research restrictions? Please check biosecurity documents and list of **select agents and toxins** (CDC): https://www.selectagents.gov/sat/list.htm | Not Applicable | |
| If you used a select agent, is the security level of the lab appropriate and reported in the manuscript? | Not Applicable | |
| If a study is subject to dual use research of concern regulations, is the name of the **authority granting approval and reference number** for the regulatory approval provided in the manuscript? | Not Applicable | |

## Reporting

The MDAR framework recommends adoption of discipline-specific guidelines, established and endorsed through community initiatives. Journals have their own policy about requiring specific guidelines and recommendations to complement MDAR.

| Adherence to community standards | Information included in the manuscript? | In which section is the information available? (Reagents and Tools Table, Materials and Methods, Figures, Data Availability Section) |
|---|---|---|
| State if relevant guidelines or checklists (e.g., **ICMJE, MIBBI, ARRIVE, PRISMA**) have been followed or provided. | Not Applicable | |
| For **tumor marker prognostic studies,** we recommend that you follow the **REMARK** reporting guidelines (see link list at top right). See author guidelines, under 'Reporting Guidelines'. Please confirm you have followed these guidelines. | Not Applicable | |
| For **phase II and III randomized controlled trials**, please refer to the **CONSORT** flow diagram (see link list at top right) and submit the CONSORT checklist (see link list at top right) with your submission. See author guidelines, under 'Reporting Guidelines'. Please confirm you have submitted this list. | Not Applicable | |

## Data Availability

| Data availability | Information included in the manuscript? | In which section is the information available? (Reagents and Tools Table, Materials and Methods, Figures, Data Availability Section) |
|---|---|---|
| Have **primary datasets** been deposited according to the journal's guidelines (see 'Data Deposition' section) and the respective accession numbers provided in the Data Availability Section? | Yes | Data Availability Section for source data |
| Were **human clinical and genomic datasets** deposited in a public access-controlled repository in accordance to ethical obligations to the patients and to the applicable consent agreement? | Not Applicable | |
| Are **computational models** that are central and integral to a study available without restrictions in a machine-readable form? Were the relevant accession numbers or links provided? | Not Applicable | |
| If publicly available data were reused, provide the respective **data citations in the reference list**. | Yes | Table S1 for source genomes |
